# Correlated Occurrence and Bypass of Frame-Shifting Insertion-Deletions (InDels) to Give Functional Proteins

**DOI:** 10.1371/journal.pgen.1003882

**Published:** 2013-10-24

**Authors:** Liat Rockah-Shmuel, Ágnes Tóth-Petróczy, Asaf Sela, Omri Wurtzel, Rotem Sorek, Dan S. Tawfik

**Affiliations:** 1 Department of Biological Chemistry, Weizmann Institute of Science, Rehovot, Israel; 2 Department of Molecular Genetics, Weizmann Institute of Science, Rehovot, Israel; Cell and Molecular Biology, Sweden

## Abstract

Short insertions and deletions (InDels) comprise an important part of the natural mutational repertoire. InDels are, however, highly deleterious, primarily because two-thirds result in frame-shifts. Bypass through slippage over homonucleotide repeats by transcriptional and/or translational infidelity is known to occur sporadically. However, the overall frequency of bypass and its relation to sequence composition remain unclear. Intriguingly, the occurrence of InDels and the bypass of frame-shifts are mechanistically related - occurring through slippage over repeats by DNA or RNA polymerases, or by the ribosome, respectively. Here, we show that the frequency of frame-shifting InDels, and the frequency by which they are bypassed to give full-length, functional proteins, are indeed highly correlated. Using a laboratory genetic drift, we have exhaustively mapped all InDels that occurred within a single gene. We thus compared the naive InDel repertoire that results from DNA polymerase slippage to the frame-shifting InDels tolerated following selection to maintain protein function. We found that InDels repeatedly occurred, and were bypassed, within homonucleotide repeats of 3–8 bases. The longer the repeat, the higher was the frequency of InDels formation, and the more frequent was their bypass. Besides an expected 8A repeat, other types of repeats, including short ones, and G and C repeats, were bypassed. Although obtained *in vitro*, our results indicate a direct link between the genetic occurrence of InDels and their phenotypic rescue, thus suggesting a potential role for frame-shifting InDels as bridging evolutionary intermediates.

## Author Summary

Homonucleotide repeats are exceptionally prone to insertions and/or deletions of bases (InDels). However, unless they occur in a multiplicity of 3 bases, InDels disrupt the reading frame and are thus expected to be purged from coding regions. Homonucleotide repeats, however, are also vulnerable to slippage by RNA polymerases and the ribosome. Using laboratory evolution techniques, we systematically mapped the occurrence of InDels within a given gene, before and after selection. Our data indicate that frame-shifting InDels were frequently bypassed to give functional proteins at surprisingly high frequencies. Further, we found a strict correlation between the repeat length, the frequency of occurrence of InDels at the DNA level, and the likelihood of bypass by transcriptional/translational slippage. Our results suggest that frame-shifting InDels might comprise functional evolutionary intermediates, and an effective mean of sequence divergence (*e.g.* when an adjacent InDel restores the frame, resulting in altered sequence and, potentially, in an altered protein structure).

## Introduction

InDels occur in all kingdoms of life, and in some organisms they are as frequent as point mutations [1]. Short sequence repeats, and homonucleotide repeats in particular, are prone to InDels due to misalignment of the DNA strands during replication (polymerase slippage) [2]. In coding regions, at least 2/3 of InDels disrupt the reading frame and are thus considered nonsense mutations leading to loss of function (for comparison, only ∼1/20 of point mutations result in a stop codon). Frame-shifting InDels are therefore thought to be tolerated only when a gene is freed from selection pressure. Indeed, under fluctuating environments, and/or within small populations, frame-shifting InDels in sequence repeats provide a rapid means of switching genes on and off [3]–[7].

Frame-shifting InDels are considered by default as nonsense mutations. There are, however, known precedents for their bypass to give functional proteins due to transcriptional and/or translational infidelity [8]–[15]. Frame-shifts are also recruited as a regulatory mechanism by ribosome programmed −/+1 frame-shifting [9]–[13]. In other cases, alternative proteins are encoded from the same gene via translational frame-shift [16], [17]. Overall, although rare, bypass of frame-shifts has been identified in organisms from all three domains of life and in viruses [9]–[11]. Most recorded events of bypass occur in long homo-adenine repeats, *e.g.* ≥10A [8], [14], but bypasses within non-repeat stretches have sporadically been reported [9], [10]. Most cases also regard explicitly evolved mechanisms for the transcriptional and/or translational bypass of frame-shifting InDels, rather than accidental slippage. It therefore remains unknown to what degree randomly occurring frame-shifting InDels can persist in coding regions under purifying selection, and whether and how the likelihood of bypass relates to the sequence contexts within which a frame-shift occurs.

Our own interest in frame-shifts followed the directed, laboratory evolution of a DNA methyltransferase M.HaeIII, towards new target DNA specificities [18]. During this process, surviving variants were identified that carried frame-shifting InDels within in their coding regions. Common to all of these variants was the location of the frame shift mutation (an ‘A’ insertion within an 8-homonucleotides repeat, Figure S1). Being unaware of the possibility of bypass, we assumed these are ‘false positives’ even though these variants did exhibit detectable level of methylation of the newly evolved target. Whilst these InDel-carrying variants disappeared in the subsequent rounds of selection, we encountered additional examples of functional variants carrying frame-shifts in the selection of other proteins. We thus became curious as to how frequent the bypass of frame-shifting InDels might be, and whether they may serve as viable evolutionary intermediates.

InDels are of particular interest as they readily create alterations in a protein's length and sequence, and thus go beyond the exchange of single side chain [19]. This is also the case with our model, M.HaeIII, is a DNA methyltransferase isolated form *Haemophilus aegyptius* that specifically methylates GGCC ds-DNA sites. M.HaeIII belongs to the prokaryotic restriction-methylation system that encompasses hundreds of different methyltransferases, each with a different DNA target specificity. The target recognition domains (TRDs) of DNA methyltransferases exhibit relatively of low structural order and are highly diverse, including extensive changes in length [20]. We suspected that the intense diversification of TRDs might relate to InDels. We used the “Path” algorithm that produces DNA sequence alignments by back-translation of known proteins sequences (Figure S2). In this manner, frame-shifting InDels that might have underlined the divergence of these sequences might be detected [21], [22]. As discussed in detail below, frame-shifting InDels were readily identified in the aligned TRDs. However, since protein evolution is assumed to occur via a series of functional intermediates [23], the evolutionary relevance of frame-shifting InDels depends on their potential to be rescued via bypass of transcriptional or translational errors.

Here, we have systematically mapped the accumulation of InDels in a laboratory-performed genetic drift of a single gene/protein. We were interested in measuring M.HaeIII's tolerance of InDels, given that, a priori, 2/3 are expected to be purged due to frame-shifts, and that the in-frame ones are also far more deleterious than point mutations [20]. To this end, we subjected M.HaeIII to iterative rounds of random mutagenesis *in-vitro* followed by purifying selection. We analyzed the gene repertoires by high-throughput sequencing; both the repertoire before selection, thus mapping the occurrence of all mutations regardless of their effect on M.HaeIII, and the repertoire after selection, thus mapping the repertoire of accepted mutations. We thereby measured, for all 987 bases along the M.HaeIII gene, the occurrence rates of InDels due to DNA polymerase slippage, and the rates of their bypass due to transcriptional/translational errors. The data indicate that the rate of bypass of frame-shifting InDels is unexpectedly high, including in relatively short homonucleotide repeats, and in repeats of nucleotides other than adenine. Foremost, we found that the propensity for the genetic occurrence of InDels, and the rates of transcriptional-translational bypass, are highly correlated.

## Results

### The laboratory drift

M.HaeIII was subjected to a laboratory genetic drift, namely to repeated rounds of random mutagenesis and purifying selection that eliminated non-functional variants (negative selection, Figure S3). To this end, M.HaeIII's gene was subjected to random mutagenesis using an error-prone DNA polymerase, at an average of 2.2±1.6 mutations per gene. The ensemble of mutated genes was ligated into an expression vector using restriction sites at the very beginning of M.HaeIII's ORF, around the ATG codon, and just after the stop codon. A plasmid vector was necessary for obtaining a large number of transformants such that large repertoires (≥10^5^ variants) could be explored. However, when driven from high copy plasmids protein, expression levels can be unrealistically high, and thus bias the level of the bypass. To minimize the levels of expression, the mutated M.HaeIII genes were cloned under the control of the tightly regulated *tet* promoter, with a constitutively expressed *tet* repressor encoded downstream. The selections throughout the drift were performed at basal expression, *i.e.*, with no inducer added to the growth media. This basal expression level was nonetheless sufficient for complete methylation of the encoding plasmid, as well as of the genome of the *E. coli* host, by wild-type M.HaeIII [18].

The ligated plasmids were transformed to *E. coli*, such that each transformed cell incorporated a different plasmid molecule carrying a different gene variant from the library of M.HaeIII mutants. In each bacterium, the transformed plasmid is replicated, and subsequently methylated at GGCC sites, or not, depending on the functionality of the M.HaeIII variant it encoded. The transformed bacteria were grown, and the plasmid pool was subsequently extracted and treated with the cognate restriction enzyme, HaeIII. Plasmids that encoded a functional M.HaeIII variant survived the digestion and thereby could be retransformed to fresh *E. coli* cells and propagated for the next round of selection [18].

We maintained ≥10^5^ transformants per round of mutagenesis-selection thus avoiding population bottlenecks and the fixation by chance of deleterious mutations. Overall, M.HaeIII's gene underwent 17 rounds of random mutagenesis and purifying selection through which the drifting population accumulated an average of 2.0±1 point mutations per gene per round.

### Systematic mapping of InDels by deep sequencing

The M.HaeIII genes encoded by the plasmid pools were subjected to high-throughput sequencing (Illumina). Sequencing was performed following the first round of random mutagenesis, thus mapping the occurrence of mutations irrespective of selection (G0, or the naive repertoire). Additionally, the pool derived after 17 rounds of mutagenesis and purifying selection as sequenced to map the repertoire of accepted mutations (G17).

The short sequencing reads (∼40 nts) were mapped to the sequence of wild-type M.HaeIII. The analyzed sequence stretch included the coding region of M.HaeIII's that was repetitively mutated and re-cloned into the selection plasmid (987 nts), as well as a plasmid region located upstream of the cloning sites that was not subjected to mutagenesis (98 nts). The latter was used to determine the background frequency of sequencing errors due to the Illumina processing, in both repertoires, G0 and G17. This background frequency was subtracted from the InDel or point mutations frequencies observed at the positions subjected to drift. This procedure allowed us to measure the mutational frequencies for all possible point mutations and InDels throughout M.HaeIII's coding region, from nucleotide position 4 (downstream the NcoI cloning site) to the stop codon (position # 993; 17 nucleotides upstream the NotI cloning site).

The frequency of a given mutation, namely a given nucleotide exchange or a given InDel, at a specific position, corresponded to the number of contigs that carried this mutation divided by the total number of sequenced contigs that covered this position. We excluded sequenced positions within contigs obtained with low accuracy score, and/or showing biases with respect to their location (*e.g.* positions located at the edges of the contigs, see Methods). In this manner, all InDels that occurred at a frequency above the background were identified, in both the naive and the selected repertoires (Table 1).

**Table 1:** Point mutations and InDels frequencies in the naive and selected repertoires (G0 and G17, respectively).

Region	Mutation type	InDel length	G0 occurrence^a^	G17 occurrence^a^
Nucleotides 4–990; open reading frame amino acids 2–330	Point mutations (S)		3×10^−3^	2×10^−2^
	InDels (I)		2.2×10^−4^	1.1×10^−4^
	S/I		16	190
	Insertions		6.7×10^−5^	1.7×10^−5^
	Deletions		1.5×10^−4^	0.9×10^−4^
	Distribution^b^	>+3	0.00%	0.03%
		+3	0.15%	0.18%
		+2	1.8%	0.4%
		+1	28.9%	9.2%
		−1	69.1%	90.2%
Nucleotides 969–990; C-terminus, amino acids 324–330	Point mutations (S)		3×10^−3^	3×10^−2^
	InDels (I)		2.0×10^−4^	0.4×10^−2^
	S/I		16	9
	Insertions		1.2×10^−5^	8.5×10^−5^
	Deletions		1.9×10^−4^	3.5×10^−3^
	Distribution^b^	+1	6.3%	1.4%
		−1	93.7%	98.6%

a The frequencies of InDels and point mutations were subtracted from the background frequencies found in the respective repertoire, G0 or G17. The latter were determined by sequencing of a 98 nts region that was not subjected to the error-prone PCR mutagenesis, and were found to be 2.1×10^−5^ for G0 and 1.6×10^−5^ for G17. The background frequencies for point mutations were found to be 1.7×10^−3^ for G0 and 8.6×10^−4^ for G17.

b The percent values relate to the fraction of a particular InDel type out of all InDels in the respective repertoire, G0 or G17.

### Mutation types and frequencies in the naive library (G0)

The *in-vitro* mutagenesis protocol applied here does not reproduce the factors that contribute to InDels formation in natural genomes. Nevertheless, certain patterns observed in the acquisition of mutations in natural genomes were also observed here. For instance, the point mutations to InDels ratio (S/I) in our unselected repertoire (G0) was found to be ∼16. This ratio is within the range observed in natural genomes (*e.g.*, ∼10 in humans, or 16 in *S. cerevisiae*) [1]. The InDels that relate to polymerase slippage in natural genomes are typically short (≤5 bp in length) with short frame-shifting InDels (*i.e.*, InDels of 1 or 2 nts) comprising over two-thirds [24]. Here, single nucleotide InDels were overwhelmingly represented (∼98% of all detected InDels; Table 1), and were thus all expected to result in frame-shift and loss of function. Finally, as detailed below, the tendency of InDels to occur within repeat regions of natural genomes is also observed in the *in-vitro* generated naive library.

### InDels occurred within repeat sequences in the naive library (G0)

The predominant mechanism generating InDels in natural genomes is polymerase slippage due to infidelity in repeat sequence pairing [2], [25], [26]. Indeed, essentially all InDels observed here occurred within homonucleotide repeats of 3–8 nucleotides (Table 2). Further, as reported for natural genomes [27]–[29], the frequency of occurrence positively correlated with repeat length (R^2^ = 0.97, Table 2).

**Table 2:** Homonucleotide repeats in M.HaeIII gene and the observed InDels frequencies within these repeats.

Homonucleotide repeats			G0		G17	
Length	nt	Occurrence^a^	Average frequency (×10^−3^)^c^	Fraction^b^	Average frequency (×10^−3^)	Fraction
8	A	1	50.25	1/1	18.04	1/1
	T	-	-	-	-	-
	C	-	-	-	-	-
	G	-	-	-	-	-
7	A	-	-	-	-	-
	T	-	-	-	-	-
	C	-	-	-	-	-
	G	-	-	-	-	-
6	A	-	-	-	-	-
	T	3	4.72	3/3	1.11	3/3
	C	-	-	-	-	-
	G	2	4.80	2/2	0.56	2/2
5	A	3	2.81	3/3	0.39	1/3
	T	2	2.95	2/2	0.76	1/2
	C	1	3.38	1/1	0.25	1/1
	G	1	2.04	1/1	0.16	0/1
4	A	12	1.93	12/12	0.15	4/12
	T	5	2.51	5/5	0.10	1/5
	C	-	-	-	-	-
	G	1	2.08	1/1	0.06	0/1
3	A	18	0.61	17/18	0.02	0/18
	T	16	0.66	15/16	0.03	0/16
	C	2	0.21	2/2	0.00	0/2
	G	3	0.50	3/3	0.03	0/3

a The number of repeats of this type as found in the nucleotide sequence of M.HaeIII's gene.

b Fraction represents the number of repeats in which InDels were found with ≥10-fold higher frequency than background out of the total number of that repeat type (*i.e.*, the occurrence of that repeat).

c The log values of the average frequencies are linearly correlated with repeat length (linear regression R^2^≥0.97; see also Figure 3).

### Frame-shifting InDels are frequently bypassed under selection (G17)

Sequencing of the selected repertoire, G17, showed that the overall tolerance of InDels was, as expected, low. Accordingly, under selection, the point mutations to InDels ratio (S/I) within M.HaeIII's coding region increased from ∼16 in G0 to ∼190 in G17 (Table 1). Frame-shifting InDels in M.HaeIII were primarily found to comprise nonsense mutations. Indeed, the purging of InDels in coding regions of natural genomes is intense [20], [30], [31].

The purging of mutations, including InDels, in disordered regions including inter-domain linkers and domain termini is far less intense than within ordered domains [20], [32]. Accordingly, the purging of InDels was >30-fold less intense at the last 7 amino acids of M.HaeIII's C-terminus. This stretch, starting from amino acid position 324, or nucleotide position 969, until the stop codon is structurally disordered and has no functional role (Table 1, lower panel, marked as ‘C-terminus, amino acids 324–330’). Thus, premature stop codons, or completely altered amino acid sequences within this region have little effect on M.HaeIII stability and function.

**Figure 1:**
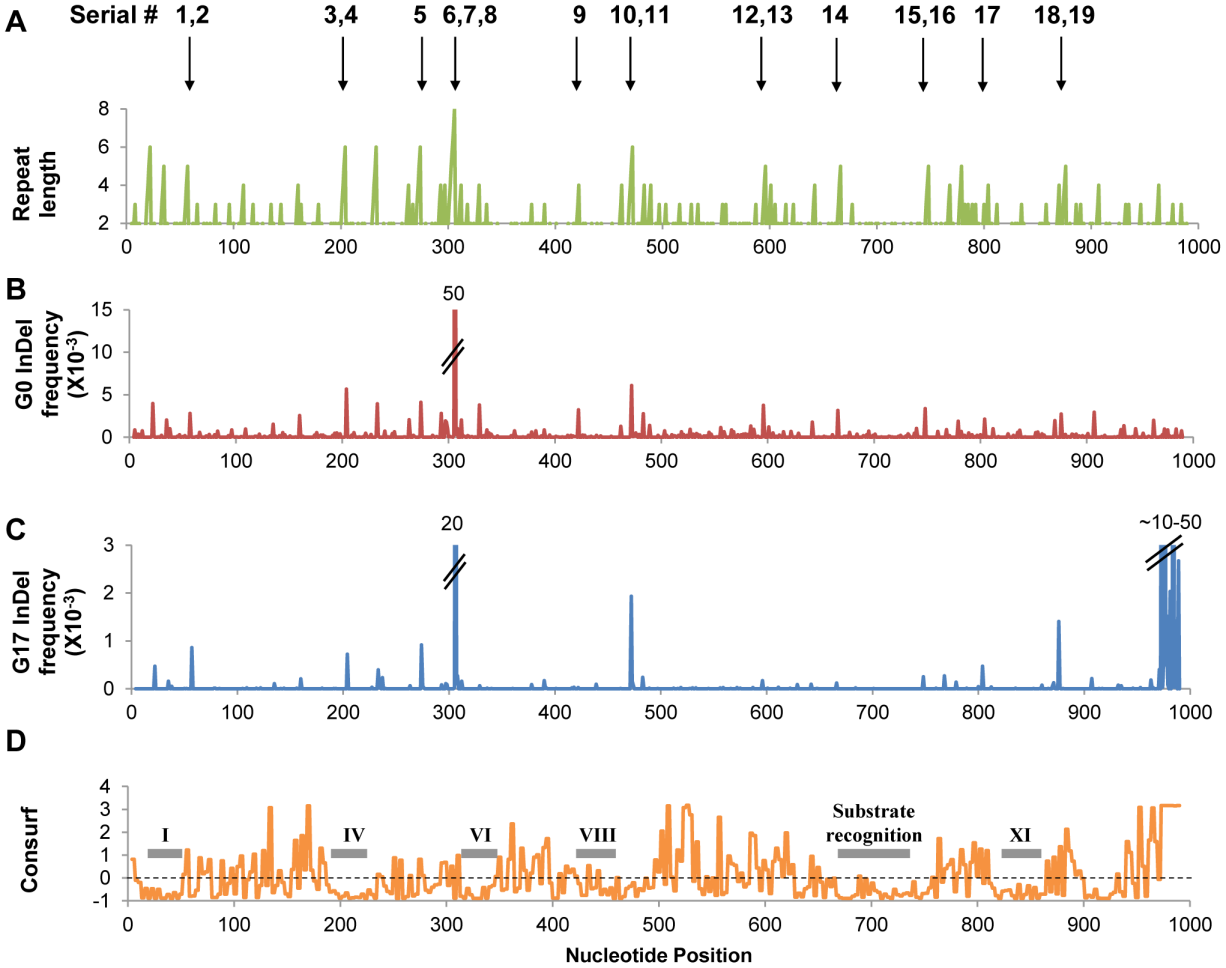
InDels frequencies in the selected (G17) and unselected (G0) repertoires. Panel A indicates the length of the homonucleotide repeat within which the InDels occurred. Panels B and C represent frequencies plotted per nucleotide position in each library. Panel D illustrates the conservation pattern in the M.HaeIII as derived by Consurf [49] – the highest the score, the higher the conservation. Also indicated are the locations of the conserved motifs shared by all DNA methyltransferases of this class (C5 methyltransferases). The serial #1–19 numbers indicate individually tested InDels (listed in Table 3).

As expected most InDels were purged, yet an expectedly high fraction was found to be tolerated. Overall, out of 337 positions in which InDels were detected in the naive repertoire, 79 positions were found to carry InDels in G17. Out of the latter, 26 positions carried InDels at a frequency ≥0.2×10^−3^ (≥10-fold higher than background frequency; Figure 1, Figure S4). Thus, InDels that were found at significant frequencies in G17 were consequently considered as potentially tolerated.

**Figure 2:**
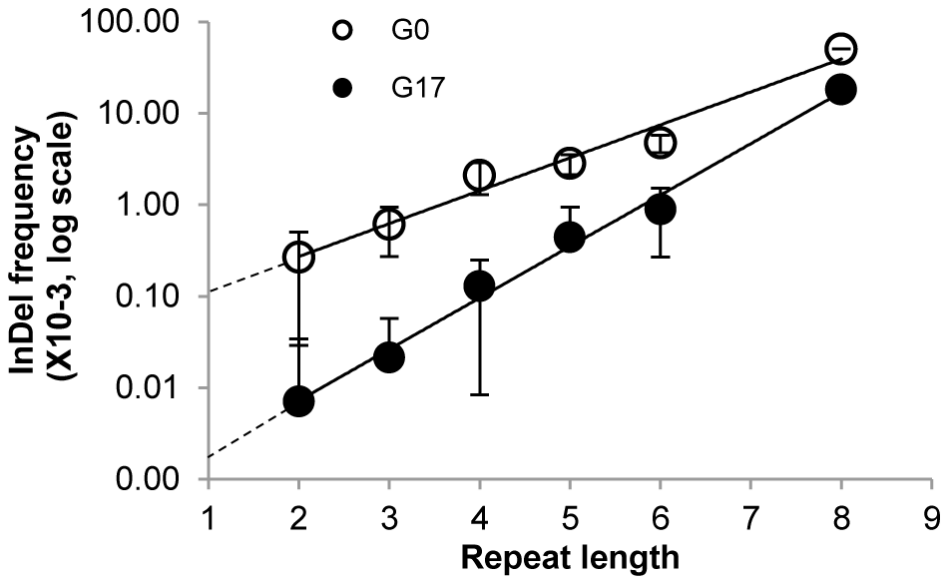
Average InDel frequencies by repeat lengths. The frequencies in the selected (G17, full circles) and in the unselected (G0, empty circles) repertoires were averaged for all InDels occurring within a given repeat length. The average frequencies, multiplied by10^3^, are presented on a log scale. Repeat length = 1 corresponds to positions whereby both flanking bases differ from the base in this position. Error bars correspond to standard deviations of the InDel frequencies for each repeat length.

As observed for the occurrence of InDels in the naive repertoire (G0), the frequencies of tolerated InDels (G17 frequencies) were highly correlated with length of the repeat in which they occurred (Table 2, Figure 2). Thus, the InDels that are most prone to occur are also the ones that are most likely to be rescued by transcriptional/translational errors. Indeed, out of 337 different positions in which InDels were identified in total (Figure S4), only 9 were observed above background rates and not within homonucleotide repeats (Table S1). These were found either at the end of homonucleotide repeats whereby the inserted or deleted nucleotide differed from the repeat one, or in short repeats such as TCTCT. These non-canonical InDels might be bypassed not at the InDel position itself, but at the adjacent repeat.

### Individually tested frame-shifting InDels

**Figure 3:**
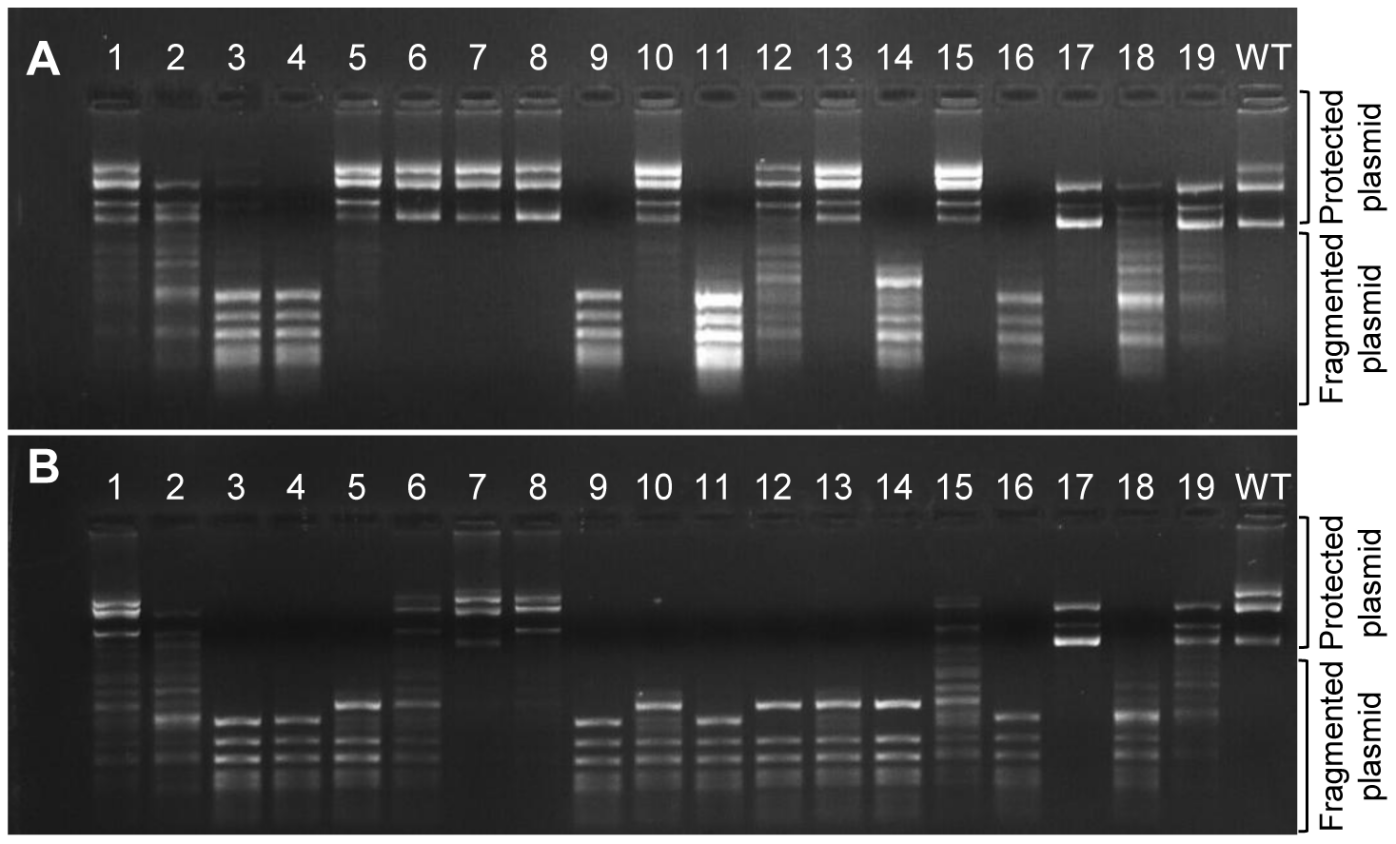
Methylation activities of M.HaeIII variants carrying individual InDels. The encoding plasmids of variants #1–19 (listed in Table 3) were extracted and the methylation activities were determined by the level of protection from digestion with HaeIII. Shown are the levels of protection for plasmids derived from *E. coli* cells grown with over-expression (with inducer; A) or with basal expression of the M.HaeIII variants (B).

To verify that the frame-shifting InDels observed under selection were indeed bypassed, we generated 15 different M.HaeIII mutants each carrying a specific InDel that had been observed in G17 at frequency above 0.2×10^−3^. We also tested four InDels identified with high frequencies in the unselected, G0 library yet with near-background frequencies in G17 (# 9, 12, 13 and 14; Figure 1, Table 3). The InDel-carrying variants were individually cloned into the same plasmid that was used for the drift and transformed into *E. coli*. Cultures derived from cells transformed with individual M.HaeIII InDel variants were grown under basal expression, or under over-expression conditions (with inducer). The functionality of individual variants was determined by the standard plasmid protection test [18], [33], *i.e.*, by the ability of the InDel-carrying variants to protect their encoding plasmids from HaeIII digestion (Figure 3). Upon over-expression, out of the 15 frame-shifted variants that corresponded to InDels found in G17, 13 showed detectable level of protection, and hence measurable methyltransferase activity (Figure 3A). Out of the four tested InDels that were found to occur in G0 but were purged under selection, two with the lowest G17 InDel frequencies (#9 and 14) showed no activity as expected. The other two (#12 and 13) exhibited detectable level of protection only when over-expressed. At basal expression level (Figure 3A), the InDels widely differ in their effects. Nonetheless, 9 out of 15 InDels were found to be bypassed at basal levels, and these also exhibited the highest G17 frequencies (Table 3). For example, variants #5–7 corresponding to InDels within the longest homorepeat (8A repeat carrying an ‘A’ deletion, or ‘A’/‘AA’ insertions, Table 3) showed high protection levels at basal expression and also the highest frequency of occurrence in G17. This result is not that surprising as long homo-A repeats are known hotspots for transcriptional/translational slippage (usually ≥10 nucleotides) [8], [14]. However, several variants carrying InDels within shorter repeats were also found to be bypassed at basal expression levels (#1, 15, 17, and 19, that occurred within 5A, 5C, 4A, and 5T homorepeats, respectively). In fact, one of these, with a deletion within a 4A repeat, seems to exhibit the highest protection level at basal expression (#17). Further, the most active InDel-carrying variants (#8 and #17) exhibited physiologically relevant levels of methylation activity as was also indicated by their ability to fully methylate their GGCC sites in the genomes of their host *E. coli* cells, even at basal expression levels (>12,000 GGCC sites protected from HaeIII digestion versus 19 in the selection plasmid, Figure S5).

**Table 3:** Individually tested InDels.

	InDel location			InDel frequency			InDel type		MTase activity^b^		
#	nt	aa	Structural location	G0 (×10^−3^)	G17 (×10^−3^)	Repeat		Sequence	Basal	OE	Conservation^c^
1	57	19	SAM binding domain -between motif I and II	2.79	0.86	5A	−A	…CCAAAA**A**GCA…	62%	78%	1.22
2							+A	…CCAAAAA**A**GCA…	13%	36%	1.22
3	204	68	Catalytic domain - motif IV	5.65	0.72	6G	−G	…TTGGGGG**G**CCGCC…	0%	0%	−0.80
4							+G	…TTGGGGGG**G**CCGCC…	0%	0%	−0.80
5	274	92	Catalytic domain - motif V	4.12	0.92	6T	−T	…AAACTTTTT**T**A…	2%	91%	0.81
6	306	102	Catalytic domain - between motif V and VI	50.25	18.04	8A	−A	…ACAAAAAAA**A**CCAA…	23%	100%	0.51
7							+A	…CAAAAAAAA**A**CCAA…	88%	100%	0.51
8							+AA	…CAAAAAAAA**AA**CCAA…	94%	100%	0.51
9	422	141	Catalytic domain - beginning of motif VIII	3.23	0.03	4T	−T	…ATATTATTT**T**GC…	0%	0%	0.08
10	472	158	End of catalytic domain (after motif VIII)	6.08	1.94	6T	−T	…GTGTTTT**T**AT…	1%	95%	−0.36
11							+T	…GTGTTTTT**T**AT…	0%	0%	−0.36
12	596	199	TRD - 25 residues before the recognition residues^a^	3.76	0.17	5A	−A	…ATAAAA**A**TAAAA…	1%	39%	−0.55
13							+A	…ATAAAAA**A**TAAAA…	5%	92%	−0.55
14	666	222	TRD - close to the recognition residues^a^	3.15	0.12	5T	−T	…ACAATTTT**T**ATG…	0%	0%	−0.01
15	748	250	TRD - 25 residues after the recognition residues^a^	3.38	0.25	5C	−C	…ACACCCC**C**AA..	32%	100%	−0.68
16							+C	…ACACCCCC**C**AA..	2%	12%	−0.68
17	804	268	TRD - before motif IX	2.14	0.47	4A	−A	…GAGGGAAA**A**GAAC	100%	100%	1.26
18	876	292	End of TRD - between motif IX and X	2.74	1.40	5T	−T	…ATTTTATTTT**T**CATT…	1%	17%	−0.70
19							+T	…ATTTTATTTTT**T**CATT…	59%	75%	−0.70

Noted within the sequence column, in bold, is the inserted base, and in bold with strikethrough the deleted one.

a The TRD recognition residues interact with the substrate DNA and are located in amino acids 219 to 244 [18], [50].

b MTase activity relates to the fraction of plasmid DNA that was completely methylated and hence protected from digestion by HaeIII; the fraction was deduced from the gel image (Figure 3) using GelAnalyzer.

c The conservation score derived from Consurf [49] using the multiple sequence alignment of M.HaeIII's orthologs with standard parameters (see Figure 1D).

Further validation that these frame-shifting InDels are bypassed to yield full length proteins was provided by a Western blot using an M.HaeIII construct that carries an epitope tag at the C-terminus (Figure S6). The observed levels of full-length proteins were well correlated with the plasmid protection levels at basal expression level, and with the G17 frequency of the InDels that these variants carry.

Although showing relatively high G17 frequencies, two variants (#3, 4, insertion and deletion of ‘G’ at 6G repeat in position 204) showed no detectible methyltransferase activity, even when over-expressed. This and the fact that some InDels are only bypassed upon over-expression, does not necessarily mean that their detection of these InDels in G17 is an artifact. Due to the short contigs of Illumina sequencing, the sequence composition of the full length drifted M.HaeIII variants within which these InDels originally occurred is unknown. They may well contain compensatory mutations at the background on which these InDels were tolerated. Indeed, laboratory drifted variants accumulate global suppressor mutations at high rates [34], as was also observed in our laboratory drift of M.HaeIII (unpublished data). In fact, the acceptance of InDels in naturally drifting sequences also seems to be correlated with the acquisition of enabling point mutations [20].

## Discussion

The relatively high frequency of tolerated InDels revealed here reinforces the possibility that frame-shifting InDels should not be considered by default as dead-ends. Rather, the sequence context within which InDels occur most frequently may also promote their tolerance. Clearly, this and other conclusions derived from this study need to be considered in view of the *in-vitro* mutagenesis protocol, the laboratory selection context, and the data that relates to one gene/protein. Nonetheless, key features that are also relevant to the acquisition of InDels in natural genomes were captured – foremost, the tendency of InDels to occur within repeat regions, and the higher frequency of frame-shifting InDels relative to in-frame ones. Our experimental system mimics these two features and thereby enabled us to systematically measure the rate of occurrence of InDels within all positions of the studied gene, and in the absence and in the presence of selection.

The levels of bypass observed in our dataset might be artificially elevated as a result of enhanced gene copy number and/or expression levels. In our experimental setup, M.HaeIII was encoded by a multi-copy plasmid and under an inducible promoter. Nonetheless, a relatively low, basal expression level (*i.e.*, without induction) was maintained due to the tight regulation of the *tet* promoter with constitutive over-expression of repressor from the same plasmid. The natural restriction-modification system from which M.HaeIII was derived is encoded by a chromosomal gene. However, whereas the restriction enzyme is tightly regulated, the methyltransferase is constitutively expressed [35], [36]. Whilst we have no direct comparison of the protein doses in nature and in our experiment, they are unlikely to differ dramatically (for comparison, a similar plasmid-based experimental setup showed no detectible GFP signal when inducer levels were ≤20 µg/ml [37], whereas in our drift no inducer was added).

### Occurrence and bypass of InDels are both correlated with repeat length

The generation of InDels in this laboratory drift was the outcome of polymerase slippage during DNA replication (components such as DNA repair were not included in the *in-vitro* replication protocol). This assumption is supported by the strict correlation between repeat lengths and frequency of InDels within their positions (Figure 2; G0 line). In non-repeat positions (positions whereby the flanking bases differ from the base in the mutated position), the occurrence frequency of InDels is close to the detection limit. Indeed, extrapolating from the observed linear correlation of repeat length and log[InDel frequency] to repeat length = 1, a frequency of ∼10^−4^ is obtained. The InDels frequency increases by ∼2.5-fold per nucleotide as the repeat length increases (Figure 2; G0).

Frame-shift bypasses are primarily associated for homo-A repeats [8], [14]. Homo-A and homo-T repeats show higher InDel frequencies than G/C repeats, but there are no clear trends regarding composition, primarily because the different base repeats are represented in M.HaeIII's gene at very different frequencies (*e.g.* 34 A/T 3-nucleotide repeats versus 5 G/C repeats; Table 2). A strict correlation was also observed between the frequency of bypassed frame-shifting InDels and repeat length. Due to the purging of most InDels by the negative/purifying selection, the slope of the correlation curve is steeper: 3.4-fold higher frequency per nucleotide length. Additionally, the intercept with the Y-axis indicates that bypass at non-repeat positions is below the background level (∼0.3×10^−5^, Figure 2; G17).

The validation of functional variants carrying individual InDels was consisted of: (*i*) their well-above background frequencies in the repertoire of M.HaeIII genes that passed the selection, G17, (*ii*) their persistence of the GGCC methylation functionality, and (iii) the detection of full-length proteins. Overall, these data suggest that the bypass of InDels occurs by slippage of the RNA polymerase and/or the ribosome, thus shifting the reading-frame either upstream or downstream (−1 or +1 shifts, respectively) to the original frame. This mechanism accounts for the observed circularity in the generation and bypass of the frame-shifting InDels. Namely, the propensity of a given sequence stretch, in this case homonucleotide repeats, towards slippage of the DNA polymerase (the genetic InDel formation), of RNA polymerase (transcriptional bypass), or of the ribosome (translational bypass), is similar.

It should, however, be noted that because frame-shifting InDels are only partly bypassed, they impose a cost. Even when bypass produces enough full-length, functional protein (*e.g.* in those cases where 100% methylation of both the plasmid and the host's chromosome are observed; Figs. 2B, S5, variants #8, 17), truncated versions derived from the original frame are also produced (Figure S6), thus producing aggregated, deleterious debris. Any advantage afforded by a frame-shifting InDel therefore depends on the benefit afforded counteracting the cost associated with such debris [38]. Nevertheless, competitions of cells carrying wild-type M.HaeIII with cells carrying different InDel variants indicated no growth inhibition by frame-shifts (Figure S7). In fact, the InDel-carrying variants unexpectedly became enriched. The toxicity associated with DNA methylation could bias growth in favor of InDel-carrying variants that are less active. However, an *E. coli* strain was used in which methylation is not toxic [39] and the most active InDel variant (#17) showed the highest growth advantage (Figure S7B). It therefore seems that, at least for the genes tested here, and within our experimental setup, the growth disadvantage imposed by frame-shifts is undetectable, possibly because expression of wild-type M.HaeIII also produces truncated fragments at comparable levels (Figure S6).

### Bypassed InDels are located between conserved motifs

The survival of frame-shifted variants was found to be dependent not only on the nucleotide repeat length, but also on the location of the InDel within the encoded protein. In accordance with previous findings [20], non-functional InDels (*e.g.* #3, #4, #9 and #14) were located within highly conserved regions, whereas the tolerated ones tend to be located between conserved motifs (Figure 1D). For example, InDels #3 and 4 comprise an insertion or deletion of a G nucleotide within a 6G repeat located in the middle of M.HaeIII's catalytic region (motif IV). These InDels were detected at high frequency in the naive repertoire, but with very low frequency in the selected, G17 repertoire (Table 3). In accordance, the variants carrying these InDels show no methylation activity, even under over-expression (Figure 3). In contrast and with agreement with the “Path” analysis for prediction for frame-shifting sites (Figure S2), InDels tolerated at high frequency tend to reside in connecting loops between conserved motifs. InDels #6–8, for example, reside in a flexible loop that connects motif V and VI (Figure S8). This tendency suggests that the bypass of most frame-shifting InDels results, as a minimum, in one point mutation. The conserved structural motifs are intolerant to substitutions and hence to InDels – to in-frame InDels [20], [40], let alone frame-shifting ones (Figure 1D, Table 3).

### Implications: ORF predictions

Altogether, our data indicate that frame-shifting InDels can be tolerated at a surprisingly high frequency. We identified at least eight readily bypassed InDels within M.HaeIII, all comprising homonucleotide repeats of 4–8 nucleotides located between the enzyme's conserved motifs. Variants carrying frame-shifting InDels within the longest 8A repeat were as expected highly tolerated, but other permissive InDels were identified in unexpected repeats, *e.g.* relatively short repeats (4 nucleotides), homo-G or -C repeats, and even next to repeats rather than within them (Table S1). The identification of multiple permissive InDels in M.HaeIII reinforces the possibility that shifted open reading frames are often read-through to give functional proteins (for examples see [8], [13], [14], [41]). The systematic mapping performed here indicated a strict correlation of the bypass likelihood with the repeat's length, and with its structural location. These parameters may assist the identification of ORFs carrying frame-shifts that may actually encode full-length, functional proteins.

### Implications: The bridging potential of frame-shifting InDels

The tolerance of a frame-shifting InDels correlates with the tendency of the position within which it occurred to acquire InDels in the first place. For the very same reason, the likelihood for reversion of an InDel, thus restoring the original frame, is very high. Reversion may occur at the same position, or at other positions within the same repeat (scenarios that are indistinguishable by sequence comparison), or, as observed here, in positions flanking the repeat (Table S1). Repeats therefore comprise hot spots for changes in length and composition, as observed in rapidly evolving proteins related to bacterial pathogenicity, or in organisms that rapidly switch on and off certain genes [3]–[7]. Similarly, the target recognition domains (TRDs) of DNA methyltransferases are highly diverse not only in sequence, but also in length [20]. Indeed, analysis of alignments of M.HaeIII and its orthologs with “Path” support the hypothesis of diversification via frame-shifting InDels (Figure S2).

The bypass of frame-shifting InDels, although transient and/or accompanied by partial loss of function, greatly increases the likelihood of occurrence of a second InDel in sequential proximity to the original one, thus restoring the frame. This may result in the diversification of both the length and composition of the entire stretch of amino acids between the two InDels, and thus, in drastic structural and functional changes occurring via functional intermediates [23]. Indeed, transcriptional and translational errors, or phenotypic mutations, may play an evolutionary role in shaping protein properties or acting as bridging intermediates [42]–[45].

## Methods

### Plasmids and strains

The M.HaeIII wild-type gene carrying four stabilizing mutations [18] was cloned with an N-terminal His-tag into pASK-IBA3+ vector (IBA, Ampicillin resistance, using NcoI and NotI; Figure S9). Plasmids were transformed into *E. coli* strain ER2267 (*Eco*K r- m- McrA- McrBC-Mrr-) in which GGCC DNA methylation is not toxic [39]. Transformants were selected by growth on ampicillin.

### Mutagenesis and selection

Random mutagenesis was performed by PCR using an error-prone polymerase (GeneMorph Mutazyme, Stratagene) and primers than flank the M.HaeIII's ORF (pASK-F and pASK-R, Table S2). The wild-type gene with 4 stabilizing mutations [18] was used as template for the first round (G0). In following rounds, the selected pool of M.HaeIII variants from the previous round was used as a template for the next one. The PCR was optimized to an average of 2.2 mutations per gene. Each round of evolution, or generation (noted as ‘G’), included the following steps (Figure S3): (*i*) The pool of M.HaeIII genes from the previous round was randomly mutated, recloned using the NcoI and NotI sites, transformed to *E. coli* and plated on agar plates containing ampicillin. (*ii*) About 10^6^ individual transformants were obtained in each round, and the cells were grown at 37°C over-night. (*iii*) The plasmid DNA was extracted and was digested with HaeIII (10–20 units, in 50 µl of NEB buffer 2, for 2 hours at 37°C). (*iv*) The plasmid DNA was purified (PCR purification kit, QIAGEN) and re-transformed for another round of enrichment. Each round of evolution included one cycle of mutagenesis and three cycles of enrichment (transformation, growth, plasmid extraction and digestion). The naive library, G0, relates to the transformed plasmid DNA derived from cloning of the repertoire of M.HaeIII genes after the first round of mutagenesis with no selection by HaeIII digestion.

### High-throughput sequencing

The samples of the naive (G0) and the selected libraries from Round 17 (G17) were prepared in the following way: (*i*) The plasmid pools were PCR amplified with primers pASKXhoI-F and pASKXhoI-R that amplified the M.HaeIII's open reading frame while appending XhoI restriction sites at both ends (Figure S9, Table S2). (*ii*) The amplified products were purified by PCR purification kit (QIAGEN) and digested with XhoI (20 units, in 60 µl of NEB buffer 4, for 2 hours at 37°C). (*iii*) The digested products were isolated by gel electrophoresis and a gel extraction kit (QIAGEN). (*iv*) To avoid bias due to poor sequencing of the edges, the fragments were ligated using the XhoI site to give concatemers. The ligation products were purified by ethanol precipitation. Sequencing libraries were prepared and sequenced according to manufacturer's protocol at the Weizmann Institute's high throughput-sequencing core facility. The obtained sequencing reads (∼40 nts) were mapped to the reference sequence of wild-type M.HaeIII with two methods: (*i*) Using NCBI blastn v2.2.20 [46] with parameters: e-value cutoff 0.0001, word size 7, and while allowing up to 6 mismatches and requiring a minimal alignment length of 24 consecutive nts, as previously described [47], [48]; and (*ii*) Using Novoalign v2.07.00 with parameters: c 4 Hash step-size 6 [47]. Point mutations, insertions and deletions were assigned based on the mapping of the sequencing reads to the reference sequence as previously described [29], [48]. Large insertions (7 or more bases) were determined by the Blast alignments, due to Blast's ability to open long gaps by performing local-alignments of the sequences. Single nucleotide mutations and short indels (7 bases or shorter) were determined by the Novoalign alignments as they take into account the base-quality information provided by the Genome Analyzer platform (using quality threshold of Q20 for filtering both indels and point mutations). Every mismatch or gap in the reads alignment relative to the wild-type reference was recorded per each nucleotide position, and further analyzed using custom Perl scripts. Only InDels that were uniformly distributed along the 40 bp reads were included. Indeed, InDels that were detected with a high bias towards the edges of reads were individually tested and found to be artifacts and were manually removed (Figure S10). InDel frequencies were determined per nucleotide position as the number of reads with a given InDel(s) divided by the total number of reads that mapped this position.

### Individual InDel variants

Individual InDels were introduced by all-around PCR using the pASK encoding wild-type M.HaeIII as template and phosphorylated primers harboring each InDel (Table S3). The PCR products were gel purified, ligated (blunt-end ligation; 10 units of T4 ligase, NEB, 2 hours at room temp.) and transformed to *E. coli*. Transformants were selected on ampicillin, and InDel incorporation was confirmed by sequencing. Appending of the C-terminal HA-tag was performed by PCR using individual InDel constructs as template and primers pASK-F and XhoCtFus-R (Table S2). The PCR products were digested with NcoI and XhoI and ligated into a modified pASK vecor containing an in-frame C-terminal HA-tag (Figure S9).

### Methylation assays

Sequence-verified InDel variants were transformed to *E. coli* and grown in LB media in the presence of ampicillin to OD_600_∼0.6. The cultures were then split: 200 ng/ml of the expression inducer anhydrotetracycline (AHT) was added to one half and the second was kept growing as is. Cultures were grown over-night at 37°C. The plasmid DNA was extracted, treated with HaeIII restriction enzyme (10–20 units, 2 hours at 37°C) and analyzed by gel electrophoresis.

## Supporting Information

Figure S1 Functional variants carrying frame-shifting InDels were identified in selections towards new target DNA specificities. As previously reported, M.HaeIII was evolved towards GGCGCC (NarI selected MTases) and GC^G^/_C_GC (TauI selected variants) DNA specificities [18]. During these selections, several functional variances emerged carrying ‘A’ insertion in the 8A nucleotide repeat. Their isolated plasmid DNAs were found to be fully methylated at the new target sites. Shown is a multiple sequence alignment of the DNA sequences of these variants and of wild-type M.HaeIII ORF. Note the ‘A’ nucleotide position 306.
(PDF)

Figure S2 The frame shifts identified in M.HaeIII orthologs by “Path”. (A) Partial alignment of *Hae*III and FnuDI GGCC methyltransferases. The two proteins can be readily aligned by back-translation of a frame shifted region that is caused by +2 insertion plus a downstream +7 insertion that restores the frame. (B) The numbers and the locations of the predicted frame shifts were mapped on M.*Hae*III protein sequence. The conserved motifs are marked in grey (dark grey – highly conserved). Strands are represented as arrows and helices as zigzags. The grey columns denote the number of the predicted frame shifts per given position. (C) Ribbon diagram of M.*Hae*III protein structure (PDB 1dct) with the DNA and the coenzyme SAM (yellow spheres) structurally aligned to M.HhaI (PDB 5mht). Frame shifted positions predicted with high probability are colored in red. Path search protocol: In order to identify possible frame shifts between M.*Hae*III homologs we used the “Path” algorithm (http://bioinfo.lifl.fr/path/index.php) [21], [22]. This program infers homologies between related protein sequences, whose divergence could be the result of both frame-shifts and point mutations. It does so by maximizing the alignment between the hypothetical back-translated DNA sequences of these proteins We used fifty-six non-redundant homologs of M.*Hae*III that were collected from REBASE database. All protein sequences were subjected to back-translational alignment using “Path”. The frame-shift penalty was reduced to −20 and all other parameters were set to default. We considered frame-shifted sequences for pairwise alignments with scores above 1000.
(PDF)

Figure S3 The laboratory genetic drift – a schematic description. M.HaeIII's open reading frame was randomly mutated by error-prone PCR. The mutated genes were cloned into the pASK vector, and the resulting plasmid library was transformed to *E. coli*. Following the first round of mutagenesis and cloning, high-throughput sequencing was performed to map the occurrence of mutations irrespective of selection (G0, or the naive repertoire). Subsequently, the plasmid library was subjected to a purifying selection. Within each transformed cell, the expressed methyltransferase variant, if active, methylated its encoding plasmid at GGCC sites and thereby protected it from digestion by the cognate, HaeIII restriction enzyme [33]. Following digestion with HaeIII, the surviving plasmids were retransformed, and subjected again to restriction for further enrichment of plasmids encoding functional methylase variants. After two cycles of enrichment (digestion and transformation), the plasmid DNA was extracted, and the surviving M.HaeIII genes were amplified and randomly mutagenized (as a pool) for the next round. The plasmid library derived from the 17^th^ round of mutagenesis and purifying selection was also subjected to high-throughput sequencing, thus mapping the repertoire of tolerated mutations (G17).
(PDF)

Figure S4 Distribution of positions in which InDels occurred according to their frequencies. The number of positions in which InDel were detected with frequencies that are 10- and 100-fold above the background frequencies. Plotted are the distributions for the naive and genetically drifted libraries (G0 and G17 respectively) and for both libraries (G0+G17).
(PDF)

Figure S5 The protection of the genomic DNA against HaeIII digestion by methylation activity of the wild-type M.HaeIII and its frame-shift carrying mutants (variants #8 and #17). Shown is the extracted chromosomal DNA of the host *E. coli* after over-night growth with plasmids carrying wild-type M.HaeIII, and mutants carrying individual frame-shifting InDels, as listed in Table 3. Bacteria were grown at the absence of the inducer (basal expression levels), genomic DNA were subsequently extracted and treated with HaeIII. In both mutants, a complete protection against HaeIII digestion was observed. WT = wild-type M.*Hae*III. The number of HaeIII sites (GGCC) in the *E. coli* genome is 12571 (based on the complete genome sequence of E. coli K-12 strain MG1655 version M52 (4,639,221 bp) [51]).
(PDF)

Figure S6 Frame shifts in M.HaeIII's gene are bypassed to produce full-length enzyme. M.HaeIII variants carrying the frame shift InDels ant C-terminal HA-tag (variants listed in Table 3) were over-expressed and their supernatants were visualized by Western blotting with monoclonal antibody against HA tag. Arrow on the left represents the expected size of a full length (as the wild-type, ‘WT’, of ∼40 KDa). Inserted – a short exposure snapshot of the wild-type M.HaeIII. Experimental protocol: Cell pellets of over-expressed InDel variants were lysed and analyzed by SDS-PAGE, electroblotted onto nitrocellulose membrane, and blocked (0.5% Tween-20 and 5% non-fat milk in 1×PBS). Tag detection was performed using polyclonal anti-HA rabbit antibodies (Abcam, diluted 1∶5000 in PBS, 0.05% Tween-20 and 5% BSA; overnight at 4°C). Peroxidase conjugated anti-rabbit secondary antibody (Jackson, 1∶10000) (0.05% Tween-20 in PBS; incubated 30 mins at 23°C) and EZ-ECL kit (Pierce) were subsequently applied and the signal was detected.
(PDF)

Figure S7 The effects of polymerase slippage on E. coli growth. (A) *E.coli* cells (of the ER2267 strain in which GGCC methylation has no toxic effects) [39] were transformed with pASK plasmids carrying wild-type M.HaeIII, or were grown with three variants carrying individual frame-shifting InDels: the most functional InDel exhibiting full methylation at basal expression (#17); a non-functional InDeled (no protection, even not when over-expressed; #3) and a variant showing partial protection under overexpression (#6; Figure 3 and Table 3). To assess the growth disadvantage associated with expressing a frame-shifting InDel, the wild-type and variant plasmids were mixed at 1∶1 ratio and transformed. The resulting cultures were grown overnight, with two subsequent serial transfers (1∶100). Plasmid DNA was extracted at four time points: the starting mixture used for transformation; transformed cells grown over-night growth (1^st^ passage); and, the two subsequent overnight cultures (2^nd^ and 3^rd^ passages). Sanger sequencing of the extracted DNA, at the relevant segment in M.HaeIII's ORF, revealed the ratio of wild-type to mutant at these four time points. As can be seen, the chromatogram of the InDel variants is shifted according to the insertion or deletion relative to the wild-type sequence. For example, in variant #3, whereas the wild-type sequence has 6 consecutive ‘G’s, in the InDel variant, only 5 ‘G’s can be found. Thus, when mixing the wild-type and InDel variant, the InDel variant's ‘C’ peak that follow the G-repeat overlaps with the wild-type's last ‘G’. (B) The ratio between the areas of the wild-type and InDel variant nucleotides' peaks reflects the frequency of the relative abundance of the wild-type and variant alleles in the grown population (in the case of variant #3 – ‘G’ for wild-type and ‘C’ for the InDel variant).
(PDF)

Figure S8 Schematic diagram of the M.HaeIII structure with the locations of the individually tested InDel variants. The Rossmann fold can be divided into the SAM-binding domain (orange, motifs I–III) and the catalytic domain that mediates the transfer the methyl group to the target DNA (light blue, motifs IV–VIII). The target recognition domain (TRD, magenta) comprises the residues important for DNA recognition at specific sequence (residues 220–243), and follows motif IX and X (the enzyme's C-termini) that interact with the Rossmann fold. The location of the tested InDel variants along the conserved motifs (I–X), and target recognition residues are noted (functional variants are noted in blue, and squares represent functional variants at basal expression levels; red squares denote non-functional variants, even at high expression levels).
(PDF)

Figure S9 The pASK plasmid map and M.HaeIII sequence. (A) Restriction map of the modified pASK vector with the HaeIII R/M sites used for the genetic drift. tetP = tet promoter; TetR = tet repressor; HisT = 6×His tag; AmpR = Ampicillin resistance gene, beta-lactamase; ColE ori = origin of replication; rbs = ribosome binding site. In blue, the NcoI/NotI restriction sites used for cloning; in red, primer locations. (B) Amino acids and DNA sequences of stabilized, His-tagged M.*Hae*III open reading frame. Note: the drifted part includes the ORF only from the NcoI site (bold red in nucleotide sequence; bold letters in the amino acids sequence). Thus, the mutations observed in the His-tag and linker part were used to determine the background mutation frequency. Amino acids numbering along the main text and supplementary were as in the wild-type sequence. Therefore, the first amino acid in the analyzed ORF (N, asparagine, in the sequence was numbered as 2). (C) The nucleotides sequence and amino acids of the HA-tag that was added at the C-terminus of individually tested variants.
(PDF)

Figure S10 Insertions with high frequencies at the edges of the reads confirmed as artifacts. Methylation activities by plasmid protection of insertions with high frequencies that were identified at the edges of reads (positions 359 and 542 nt alongside other individual InDel variants (listed in Table 3). Variants were tested following basal expression (A) or over-expression (B). The encoding plasmids DNA were extracted and the methylation activities were measured by the level of protection from digestion by HaeIII.
(PDF)

Table S1 InDels within non-repeat sequences. Noted within the sequence column, in bold, is the inserted base, and in bold with strikethrough the deleted one.
(PDF)

Table S2 Primers list used for library preparation and sequencing. Underlined – the introduced XhoI site. ^a^ The priming site is in accordance with the plasmid map (Figure S9).
(PDF)

Table S3 Primers list used for all around PCR generating individually tested InDels (described in Table 3).
(PDF)
