# Supplementary material for: Correlated Occurrence and Bypass of Frame-Shifting Insertion-Deletions (InDels) to Give Functional Proteins
Source: PLoS Genet. 2013 Oct 24;9(10):e1003882. doi: 10.1371/journal.pgen.1003882 (PMC3812077; doi:10.1371/journal.pgen.1003882)
Supplement: Figure S1 — Functional variants carrying frame-shifting InDels were identified in selections towards new target DNA specificities. As previously reported, M.HaeIII was evolved towards GGCGCC (NarI selected MTases) and GCG/CGC (TauI selected variants) DNA specificities [18]. During these selections, several functional variances emerged carrying ‘A’ insertion in the 8A nucleotide repeat. Their isolated plasmid DNAs were found to be fully methylated at the new target sites. Shown is a multiple sequence alignment of the DNA sequences of these variants and of wild-type M.HaeIII ORF. Note the ‘A’ nucleotide position 306. (PDF) [file pgen.1003882.s001.pdf]

```
                    10        20        30        40        50        60        70        80        90       100
           ....|....|....|....|....|....|....|....|....|....|....|....|....|....|....|....|....|....|....|....|
WT_MHaeIII GCAAATTTAATTAGTCTTTTTTCAGGTGCAGGGGGATTAGATCTTGGCTTCCAAAAAGCAGGATTTCGTATTATTGCCGCAAATGAATATGATAAATCAA
NarG8005   ..................................................................................G.................
NarG8009   .T.........................................................................A........................
NarG9003   .T...............C..................................................................................
TauG8002   ....G...............................................................................................
TauG8005   ....................................................................................................
TauG9001   .T..........................................C.......................................................
TauG9006   ............................................................................T.......................
TauG9008   .T..........................................................................T.......................
TauG10001  .T.........................................................................T........................
TauG10007  ............................................................................T.......................

                   110       120       130       140       150       160       170       180       190       200
           ....|....|....|....|....|....|....|....|....|....|....|....|....|....|....|....|....|....|....|....|
WT_MHaeIII TATGGAAAACATATGAAAGTAATCATTCAGCTAAATTGATCAAAGGCGATATATCTAAAATTTCTTCAGATGAATTCCCTAAGTGTGATGGAATTATTGG
NarG8005   ....................................................................................................
NarG8009   .............................A......................................................................
NarG9003   .............................................................C......................................
TauG8002   .............C......................................................................................
TauG8005   ....................................................................................................
TauG9001   .............C................................T..............C......................................
TauG9006   ....................................................................................................
TauG9008   .............................A......................................................................
TauG10001  ..........T........................................................T................................
TauG10007  ....................................................................................................

                   210       220       230       240       250       260       270       280       290       300
           ....|....|....|....|....|....|....|....|....|....|....|....|....|....|....|....|....|....|....|....|
WT_MHaeIII GGGGCCGCCTTGTCAATCTTGGAGTGAGGGGGGATCTCTTAGAGGAATTGATGATCCTCGGGGCAAACTTTTTTATGAATATATTCGGATTTTAAAACAA
NarG8005   ....................................................................................................
NarG8009   ......................................A.............................................................
NarG9003   .........A................T.........................................................................
TauG8002   ..............................T.....................................................................
TauG8005   ....................................................................................................
TauG9001   ....................................................................................C...............
TauG9006   ......................................A.........C...............................................C...
TauG9008   ....................................................................................................
TauG10001  ......................................A.........C...............................................C...
TauG10007  ......................................A.........C...............................................C...

                   310       320       330       340       350       360       370       380       390       400
           ....|....|....|....|....|....|....|....|....|....|....|....|....|....|....|....|....|....|....|....|
WT_MHaeIII AAAAAA-CCAAAATTCTTTCTTGCCGAAAACGTTAAAGGAATGCTGGCTCAGCGTCATAATAAGGCTGTTCAAGAATTTATCCAAGAATTTGATAATGCT
NarG8005   ......A.............................................................................................
NarG8009   ......A...........................................................C..........................C......
NarG9003   ......A......................................T..............................................T.......
TauG8002   ......AA.............C......................................A.......................................
TauG8005   ......A....T......................................................C.................................
TauG9001   ......A....T................................................A.......................................
TauG9006   ......A..............C..............................................................................
TauG9008   ......A....T................................................A.......................................
TauG10001  ......A..............C.....................................................................C.........
TauG10007  ......A..............C..............................................................................

                   410       420       430       440       450       460       470       480       490       500
           ....|....|....|....|....|....|....|....|....|....|....|....|....|....|....|....|....|....|....|....|
WT_MHaeIII GGATATGATGTCCATATTATTTTGCTTAATGCGAATGATTATGGTGTAGCTCAAGATAGAAAACGTGTTTTTTATATTGGTTTTAGAAAAGAGTTAAATA
NarG8005   ....................................................................................................
NarG8009   ....................................................................................................
NarG9003   ....................................................................................................
TauG8002   ....................................................................................................
TauG8005   ....................................................................................................
TauG9001   ....................................................................................................
TauG9006   ....................................................................................................
TauG9008   ....................................................................................................
TauG10001  ....................................................................................................
TauG10007  ....................................................................................................

                   510       520       530       540       550       560       570       580       590       600
           ....|....|....|....|....|....|....|....|....|....|....|....|....|....|....|....|....|....|....|....|
WT_MHaeIII TAAATTATCTTCCACCCATTCCACATTTGATAAAGCCAACATTGAAGGACGTCATTTGGGATCTTAAGGATAATCCAATTCCAGCTTTAGATAAAAATAA
NarG8005   ....................................................................................................
NarG8009   ....................................................................................................
NarG9003   ....................................................................................................
TauG8002   ....................................................................................................
TauG8005   ....................................................................................................
TauG9001   .....................................................................................A..............
TauG9006   ...............................................................................C....................
TauG9008   ....................................................................................................
TauG10001  ....................................................................................................
TauG10007  ...............................................................................C....................

                   610       620       630       640       650       660       670       680       690       700
           ....|....|....|....|....|....|....|....|....|....|....|....|....|....|....|....|....|....|....|....|
WT_MHaeIII AACAAATGGTAATAAATGTATTTATCCTAATCACGAATATTTTATAGGATCATATTCAACAATTTTTATGAGTAGAAATCGTGTTAGACAATGGAATGAA
NarG8005   .........................................................................G..........C...............
```

```
NarG8009    ...........................................................................T........................
NarG9003    .........................................................................G..........................
TauG8002    .........................................................................G..........................
TauG8005    ......................C..................................................G..........................
TauG9001    .........................................................................G.............G............
TauG9006    .........................................................................G.............G............
TauG9008    ...............................................................A.........G.............G............
TauG10001   .........................................................................G..........................
TauG10007   .........................................................................G.............G............

                    710       720       730       740       750       760       770       780       790       800
            ....|....|....|....|....|....|....|....|....|....|....|....|....|....|....|....|....|....|....|....|
WT_MHaeIII  CCAGCATTCACAGTTCAAGCATCTGGACGACAATGTCAATTACACCCCCAAGCTCCTGTAATGTTAAAAGTTAGTAAAAATTTAAATAAATTTGTTGAGG
NarG8005    .......................C.................................................A..........T...............
NarG8009    ............A.......................................................................T...............
NarG9003    ....................................................................................T...............
TauG8002    ..................................................G.................................T...............
TauG8005    ....................................................................................T...............
TauG9001    .................................................................................A..T...............
TauG9006    .................................................................................A..T...............
TauG9008    .................................................................................A..T...............
TauG10001   ......................................G.............................................T...............
TauG10007   .................................................................................A..T...............

                    810       820       830       840       850       860       870       880       890       900
            ....|....|....|....|....|....|....|....|....|....|....|....|....|....|....|....|....|....|....|....|
WT_MHaeIII  GAAAAGAACATTTATATCGCAGACTAACGGTTCGGGAATGTGCACGAGTGCAAGGATTTCCAGATGATTTTATTTTTCATTACGAAAGTTTGAATGATGG
NarG8005    ....................................................................................................
NarG8009    ...............................................T....................................................
NarG9003    ...............................................T....................................................
TauG8002    ...............................................T....................................................
TauG8005    ........T...............................C...........................................................
TauG9001    ........................................................................................A...........
TauG9006    ...............................................T....................................................
TauG9008    ........................................................................................A...........
TauG10001   ...............................................T....................................................
TauG10007   ...............................................T....................................................

                    910       920       930       940       950       960       970       980       990
            ....|....|....|....|....|....|....|....|....|....|....|....|....|....|....|....|....|....|....|....
WT_MHaeIII  TTATAAAATGATCGGTAACGCTGTGCCTGTAAATTTAGCATATGAAATAGCTAAGACAATAAAATCTGCATTGGAAATTCGTAAAGGTAATTAG
NarG8005    ..............................................................................................
NarG8009    ...........................C..................................................................
NarG9003    C..........................................................................T..................
TauG8002    ..............................................................................................
TauG8005    ..............................................................................................
TauG9001    .............................................................................C................
TauG9006    ..............................................................................................
TauG9008    ........................................................GC....................................
TauG10001   .......................................T......T...............................................
TauG10007   ..............................................................................................
```
