# Supplementary material for: Correlated Occurrence and Bypass of Frame-Shifting Insertion-Deletions (InDels) to Give Functional Proteins
Source: PLoS Genet. 2013 Oct 24;9(10):e1003882. doi: 10.1371/journal.pgen.1003882 (PMC3812077; doi:10.1371/journal.pgen.1003882)
Supplement: Figure S2 — The frame shifts identified in M.HaeIII orthologs by “Path”. (A) Partial alignment of HaeIII and FnuDI GGCC methyltransferases. The two proteins can be readily aligned by back-translation of a frame shifted region that is caused by +2 insertion plus a downstream +7 insertion that restores the frame. (B) The numbers and the locations of the predicted frame shifts were mapped on M.HaeIII protein sequence. The conserved motifs are marked in grey (dark grey – highly conserved). Strands are represented as arrows and helices as zigzags. The grey columns denote the number of the predicted frame shifts per given position. (C) Ribbon diagram of M.HaeIII protein structure (PDB 1dct) with the DNA and the coenzyme SAM (yellow spheres) structurally aligned to M.HhaI (PDB 5mht). Frame shifted positions predicted with high probability are colored in red. Path search protocol: In order to identify possible frame shifts between M.HaeIII homologs we used the “Path” algorithm (http://bioinfo.lifl.fr/path/index.php) [21], [22]. This program infers homologies between related protein sequences, whose divergence could be the result of both frame-shifts and point mutations. It does so by maximizing the alignment between the hypothetical back-translated DNA sequences of these proteins We used fifty-six non-redundant homologs of M.HaeIII that were collected from REBASE database. All protein sequences were subjected to back-translational alignment using “Path”. The frame-shift penalty was reduced to −20 and all other parameters were set to default. We considered frame-shifted sequences for pairwise alignments with scores above 1000. (PDF) [file pgen.1003882.s002.pdf]

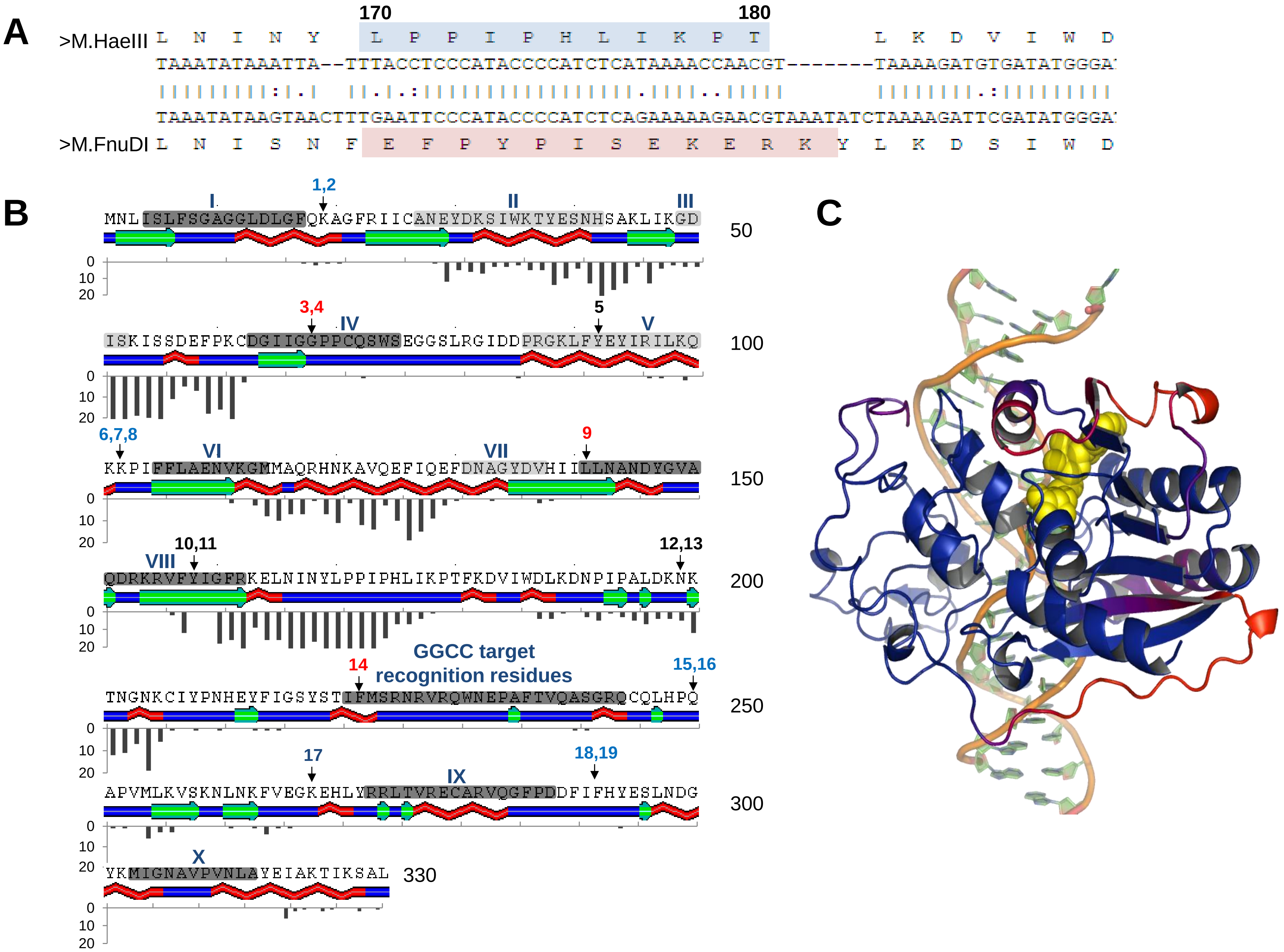
A
170
180
>M.HaeIII L N I N Y L P P I P H L I K P T L K D V I W D
TAAATATAAATTA--TTTACCTCCCATACCCCATCTCATAAAACCAACGT-------TAAAAGATGTGATATGGGA
TAAATATAAGTAACTTTGAATTCCCATACCCCATCTCAGAAAAAGAACGTAAATATCTAAAAGATTCGATATGGGA
>M.FnuDI L N I S N F E F P Y P I S E K E R K Y L K D S I W D
B
I
1,2
II
III
MNLISLFSGAGGLDLGFQKAGFRIICANEYDKSIWKTYESNHSAKLIKGD
50
3,4
IV
5
V
ISKISSDEFPKCDGIIGGPPCQSWSEGGSLRGIDDPRGKLFYEYIRILKQ
100
6,7,8
VI
VII
9
KKPIFFLAENVKGMMAQRHNKAVQEFIQEFDNAGYDVHIILLNANDYGVA
150
VIII
10,11
12,13
QDRKRVFYIGFRKELNINYLPPIPHLIKPTFKDVIWDLKDNPIPALDKNK
200
GGCC target recognition residues
14
15,16
TNGNKCIYPNHEYFIGSYSTIFMSRNRVRQWNEPAFTVQASGRQCQLHPQ
250
17
18,19
IX
APVMLKVSKNLNKFVEGKEHLYRRLTVRECARVQGFPDDFIFHYESLNDG
300
X
YKMIGNAVPVNLAYEIAKTIKSAL
330
0
10
20
C
