# Supplementary figures and images for: Correlated Occurrence and Bypass of Frame-Shifting Insertion-Deletions (InDels) to Give Functional Proteins
Source: PLoS Genet. 2013 Oct 24;9(10):e1003882. doi: 10.1371/journal.pgen.1003882 (PMC3812077; doi:10.1371/journal.pgen.1003882)

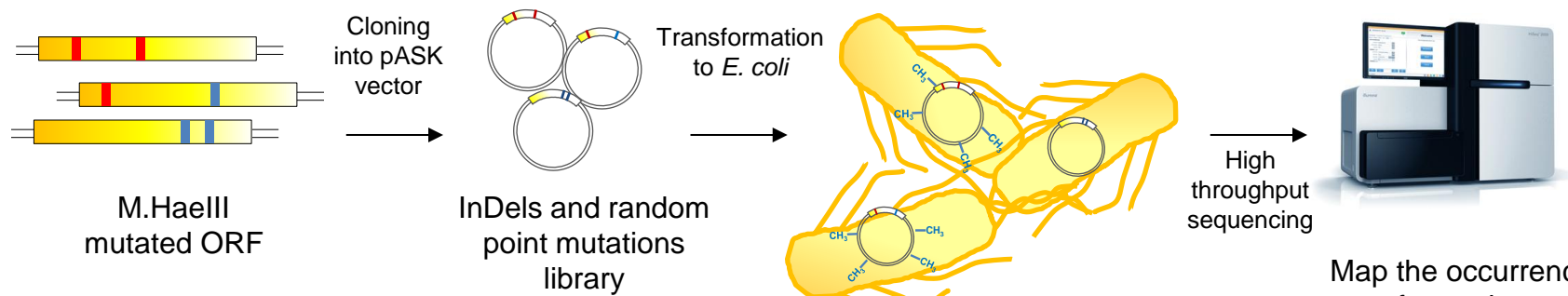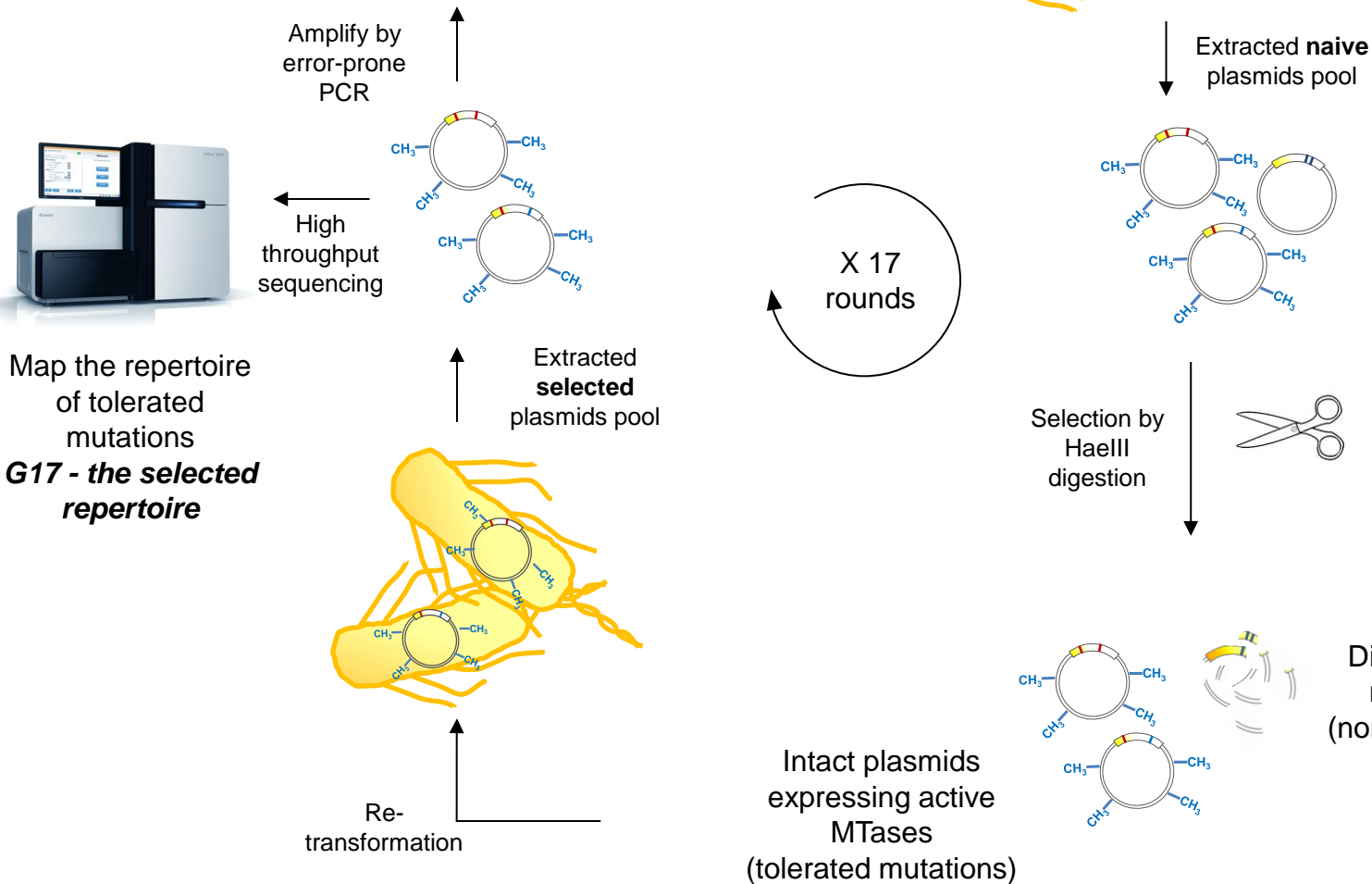

Supplement: Figure S3 — The laboratory genetic drift – a schematic description. M.HaeIII's open reading frame was randomly mutated by error-prone PCR. The mutated genes were cloned into the pASK vector, and the resulting plasmid library was transformed to E. coli. Following the first round of mutagenesis and cloning, high-throughput sequencing was performed to map the occurrence of mutations irrespective of selection (G0, or the naive repertoire). Subsequently, the plasmid library was subjected to a purifying selection. Within each transformed cell, the expressed methyltransferase variant, if active, methylated its encoding plasmid at GGCC sites and thereby protected it from digestion by the cognate, HaeIII restriction enzyme [33]. Following digestion with HaeIII, the surviving plasmids were retransformed, and subjected again to restriction for further enrichment of plasmids encoding functional methylase variants. After two cycles of enrichment (digestion and transformation), the plasmid DNA was extracted, and the surviving M.HaeIII genes were amplified and randomly mutagenized (as a pool) for the next round. The plasmid library derived from the 17th round of mutagenesis and purifying selection was also subjected to high-throughput sequencing, thus mapping the repertoire of tolerated mutations (G17). (PDF) [file pgen.1003882.s003.pdf]

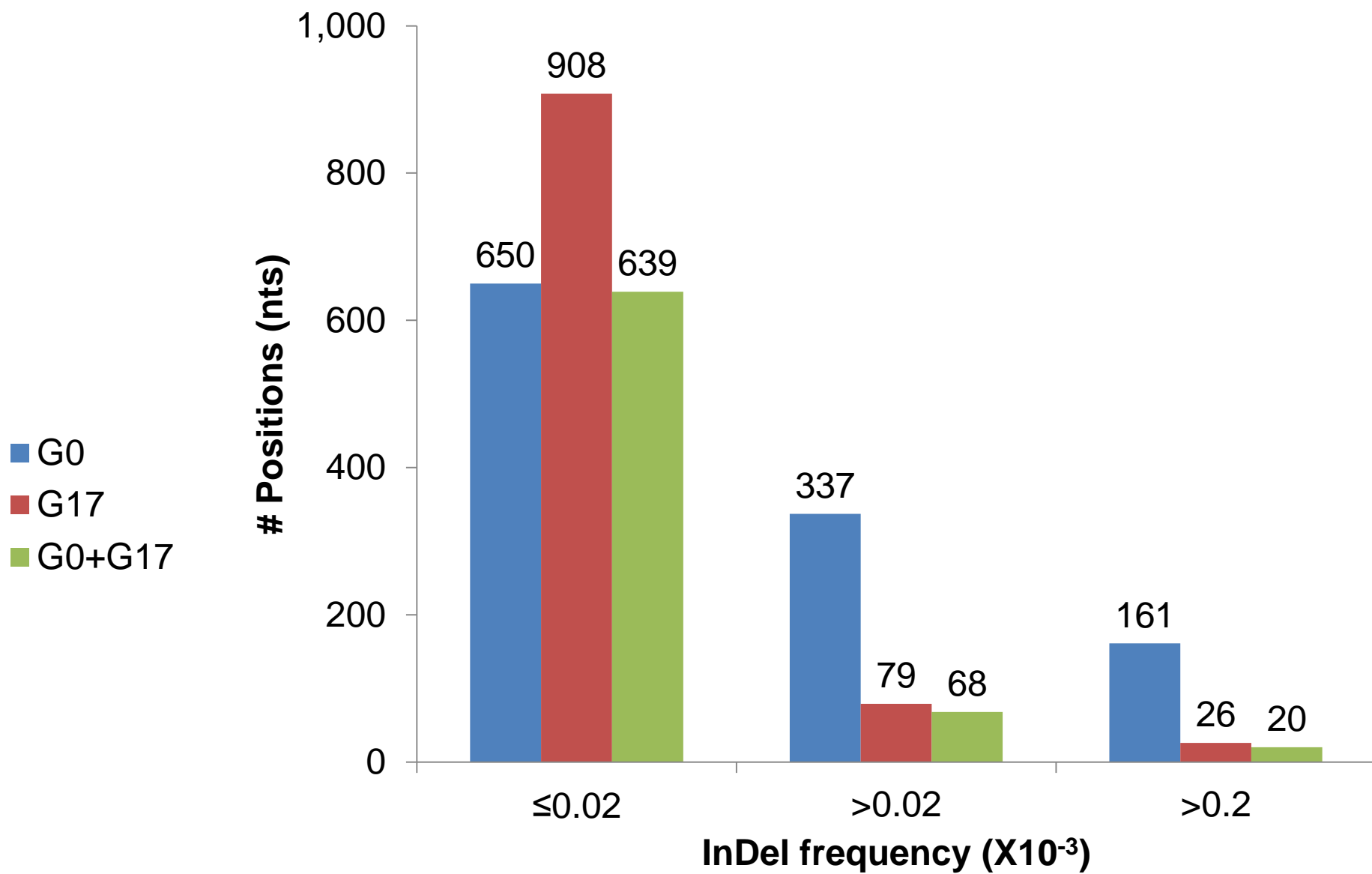

Supplement: Figure S4 — Distribution of positions in which InDels occurred according to their frequencies. The number of positions in which InDel were detected with frequencies that are 10- and 100-fold above the background frequencies. Plotted are the distributions for the naive and genetically drifted libraries (G0 and G17 respectively) and for both libraries (G0+G17). (PDF) [file pgen.1003882.s004.pdf]

No  
plasmid

Wild-  
type

#17

#8

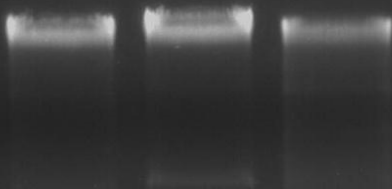

Supplement: Figure S5 — The protection of the genomic DNA against HaeIII digestion by methylation activity of the wild-type M.HaeIII and its frame-shift carrying mutants (variants #8 and #17). Shown is the extracted chromosomal DNA of the host E. coli after over-night growth with plasmids carrying wild-type M.HaeIII, and mutants carrying individual frame-shifting InDels, as listed in Table 3. Bacteria were grown at the absence of the inducer (basal expression levels), genomic DNA were subsequently extracted and treated with HaeIII. In both mutants, a complete protection against HaeIII digestion was observed. WT = wild-type M.HaeIII. The number of HaeIII sites (GGCC) in the E. coli genome is 12571 (based on the complete genome sequence of E. coli K-12 strain MG1655 version M52 (4,639,221 bp) [51]). (PDF) [file pgen.1003882.s005.pdf]

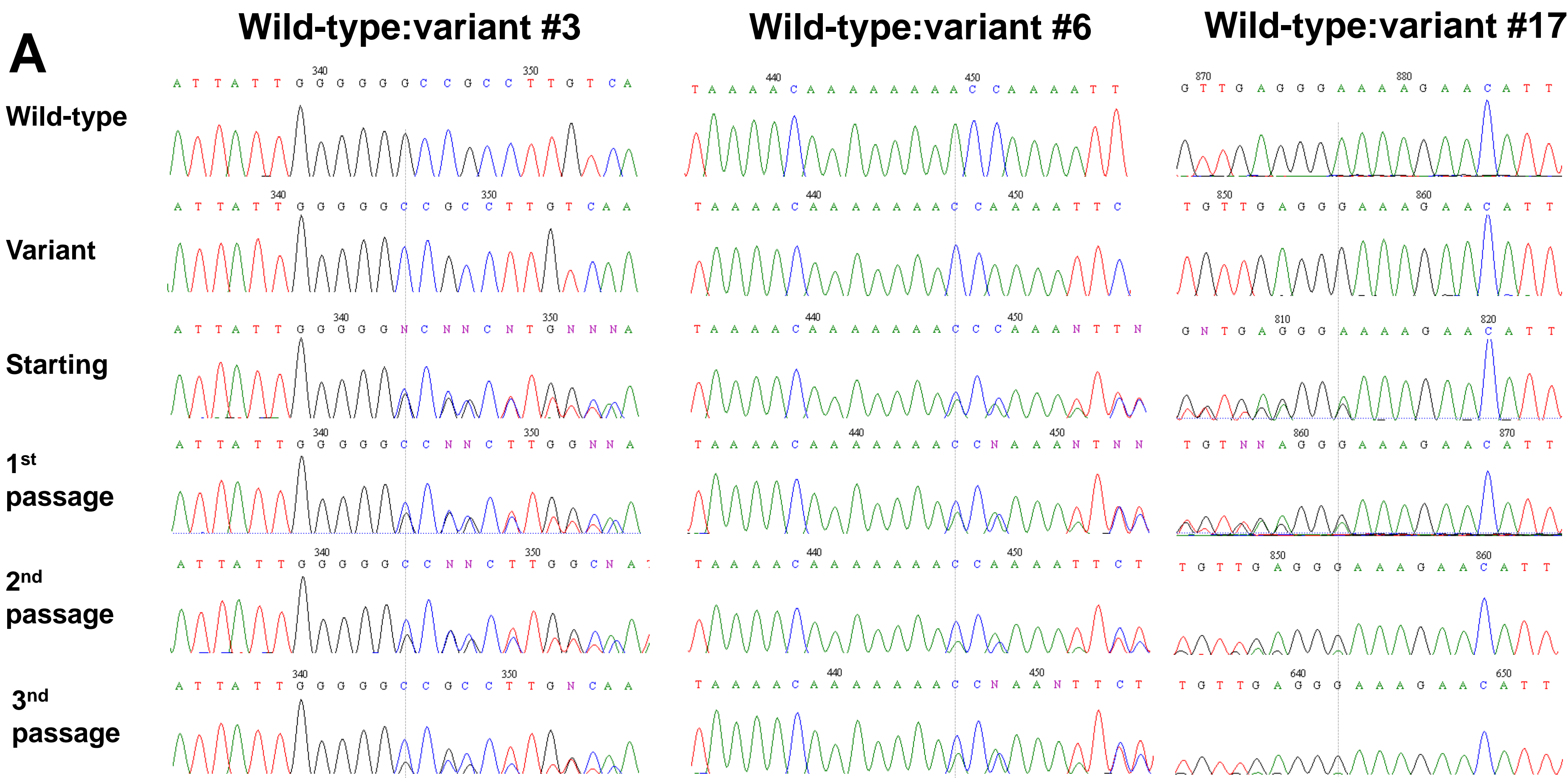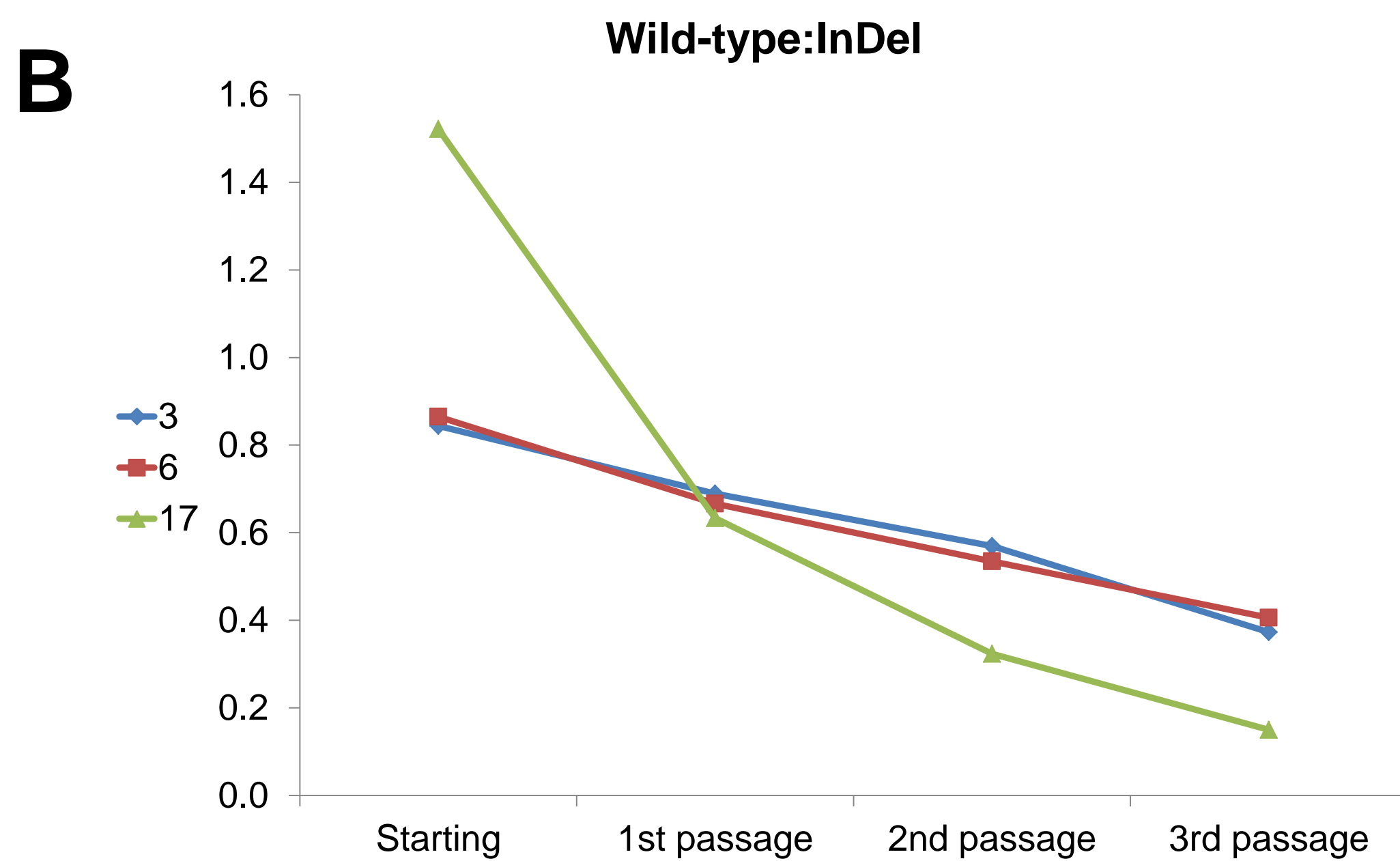

Supplement: Figure S7 — The effects of polymerase slippage on E. coli growth. (A) E.coli cells (of the ER2267 strain in which GGCC methylation has no toxic effects) [39] were transformed with pASK plasmids carrying wild-type M.HaeIII, or were grown with three variants carrying individual frame-shifting InDels: the most functional InDel exhibiting full methylation at basal expression (#17); a non-functional InDeled (no protection, even not when over-expressed; #3) and a variant showing partial protection under overexpression (#6; Figure 3 and Table 3). To assess the growth disadvantage associated with expressing a frame-shifting InDel, the wild-type and variant plasmids were mixed at 1∶1 ratio and transformed. The resulting cultures were grown overnight, with two subsequent serial transfers (1∶100). Plasmid DNA was extracted at four time points: the starting mixture used for transformation; transformed cells grown over-night growth (1st passage); and, the two subsequent overnight cultures (2nd and 3rd passages). Sanger sequencing of the extracted DNA, at the relevant segment in M.HaeIII's ORF, revealed the ratio of wild-type to mutant at these four time points. As can be seen, the chromatogram of the InDel variants is shifted according to the insertion or deletion relative to the wild-type sequence. For example, in variant #3, whereas the wild-type sequence has 6 consecutive ‘G’s, in the InDel variant, only 5 ‘G’s can be found. Thus, when mixing the wild-type and InDel variant, the InDel variant's ‘C’ peak that follow the G-repeat overlaps with the wild-type's last ‘G’. (B) The ratio between the areas of the wild-type and InDel variant nucleotides' peaks reflects the frequency of the relative abundance of the wild-type and variant alleles in the grown population (in the case of variant #3 – ‘G’ for wild-type and ‘C’ for the InDel variant). (PDF) [file pgen.1003882.s007.pdf]

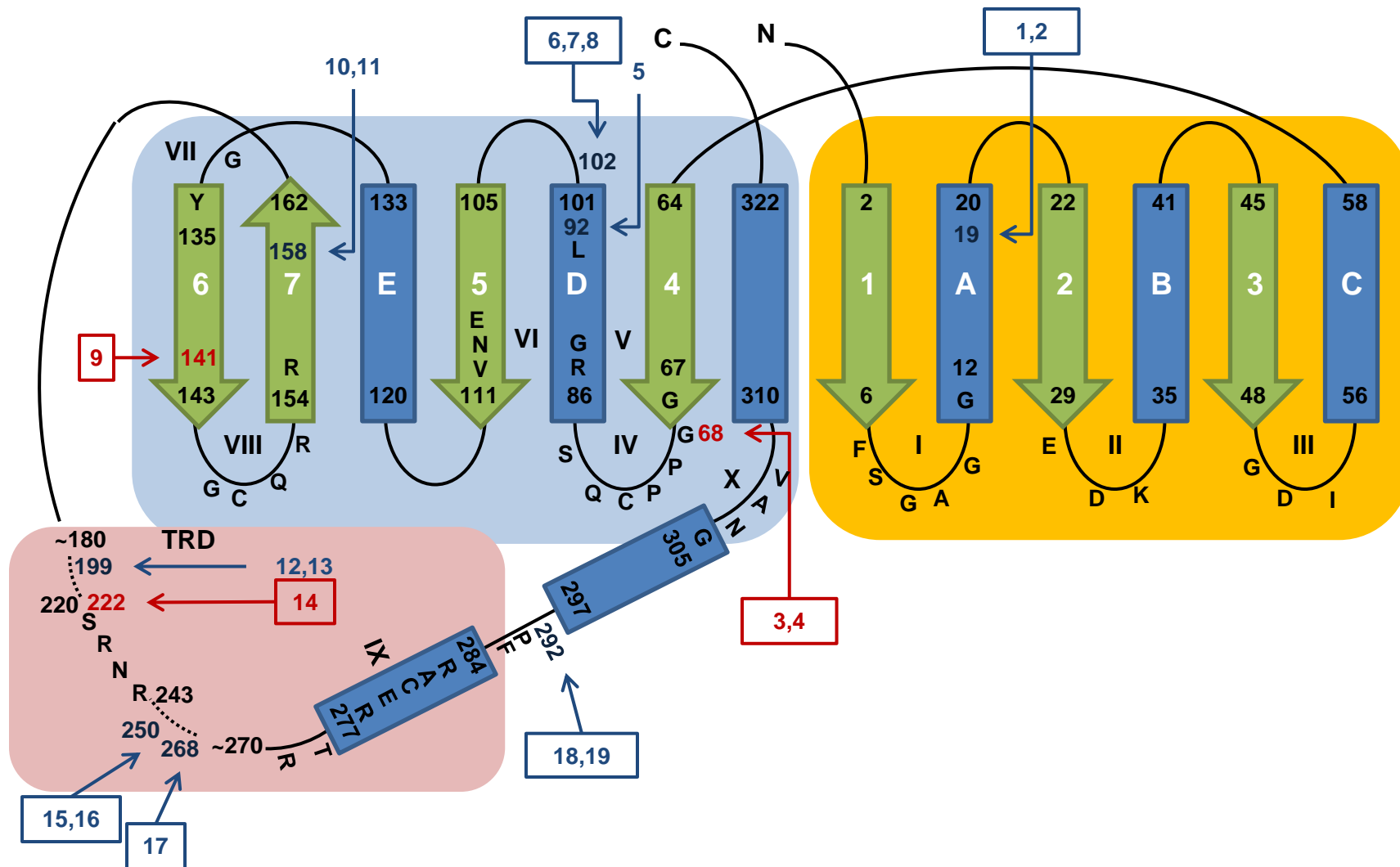

Supplement: Figure S8 — Schematic diagram of the M.HaeIII structure with the locations of the individually tested InDel variants. The Rossmann fold can be divided into the SAM-binding domain (orange, motifs I–III) and the catalytic domain that mediates the transfer the methyl group to the target DNA (light blue, motifs IV–VIII). The target recognition domain (TRD, magenta) comprises the residues important for DNA recognition at specific sequence (residues 220–243), and follows motif IX and X (the enzyme's C-termini) that interact with the Rossmann fold. The location of the tested InDel variants along the conserved motifs (I–X), and target recognition residues are noted (functional variants are noted in blue, and squares represent functional variants at basal expression levels; red squares denote non-functional variants, even at high expression levels). (PDF) [file pgen.1003882.s008.pdf]

**A**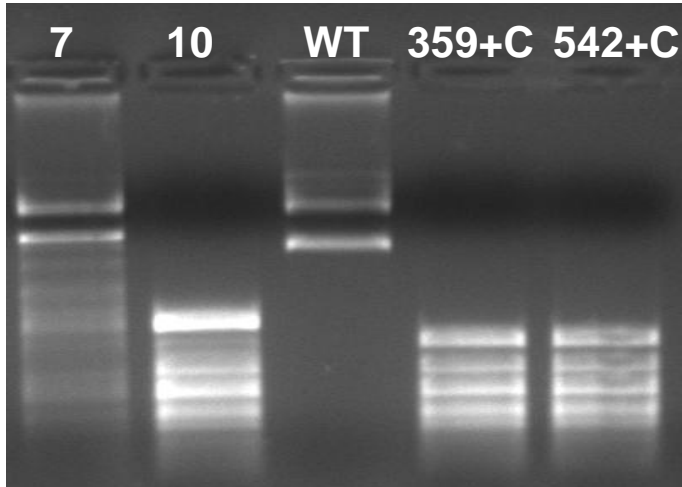**B**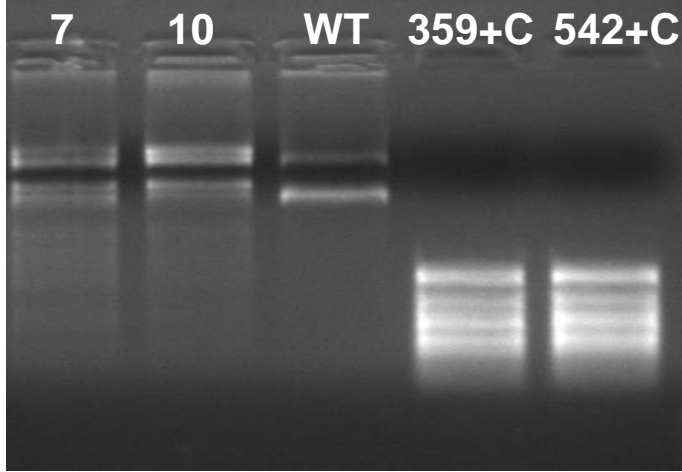

Supplement: Figure S10 — Insertions with high frequencies at the edges of the reads confirmed as artifacts. Methylation activities by plasmid protection of insertions with high frequencies that were identified at the edges of reads (positions 359 and 542 nt alongside other individual InDel variants (listed in Table 3). Variants were tested following basal expression (A) or over-expression (B). The encoding plasmids DNA were extracted and the methylation activities were measured by the level of protection from digestion by HaeIII. (PDF) [file pgen.1003882.s010.pdf]
