# Supplementary material for: Correlated Occurrence and Bypass of Frame-Shifting Insertion-Deletions (InDels) to Give Functional Proteins
Source: PLoS Genet. 2013 Oct 24;9(10):e1003882. doi: 10.1371/journal.pgen.1003882 (PMC3812077; doi:10.1371/journal.pgen.1003882)
Supplement: Figure S9 — The pASK plasmid map and M.HaeIII sequence. (A) Restriction map of the modified pASK vector with the HaeIII R/M sites used for the genetic drift. tetP = tet promoter; TetR = tet repressor; HisT = 6×His tag; AmpR = Ampicillin resistance gene, beta-lactamase; ColE ori = origin of replication; rbs = ribosome binding site. In blue, the NcoI/NotI restriction sites used for cloning; in red, primer locations. (B) Amino acids and DNA sequences of stabilized, His-tagged M.HaeIII open reading frame. Note: the drifted part includes the ORF only from the NcoI site (bold red in nucleotide sequence; bold letters in the amino acids sequence). Thus, the mutations observed in the His-tag and linker part were used to determine the background mutation frequency. Amino acids numbering along the main text and supplementary were as in the wild-type sequence. Therefore, the first amino acid in the analyzed ORF (N, asparagine, in the sequence was numbered as 2). (C) The nucleotides sequence and amino acids of the HA-tag that was added at the C-terminus of individually tested variants. (PDF) [file pgen.1003882.s009.pdf]

A

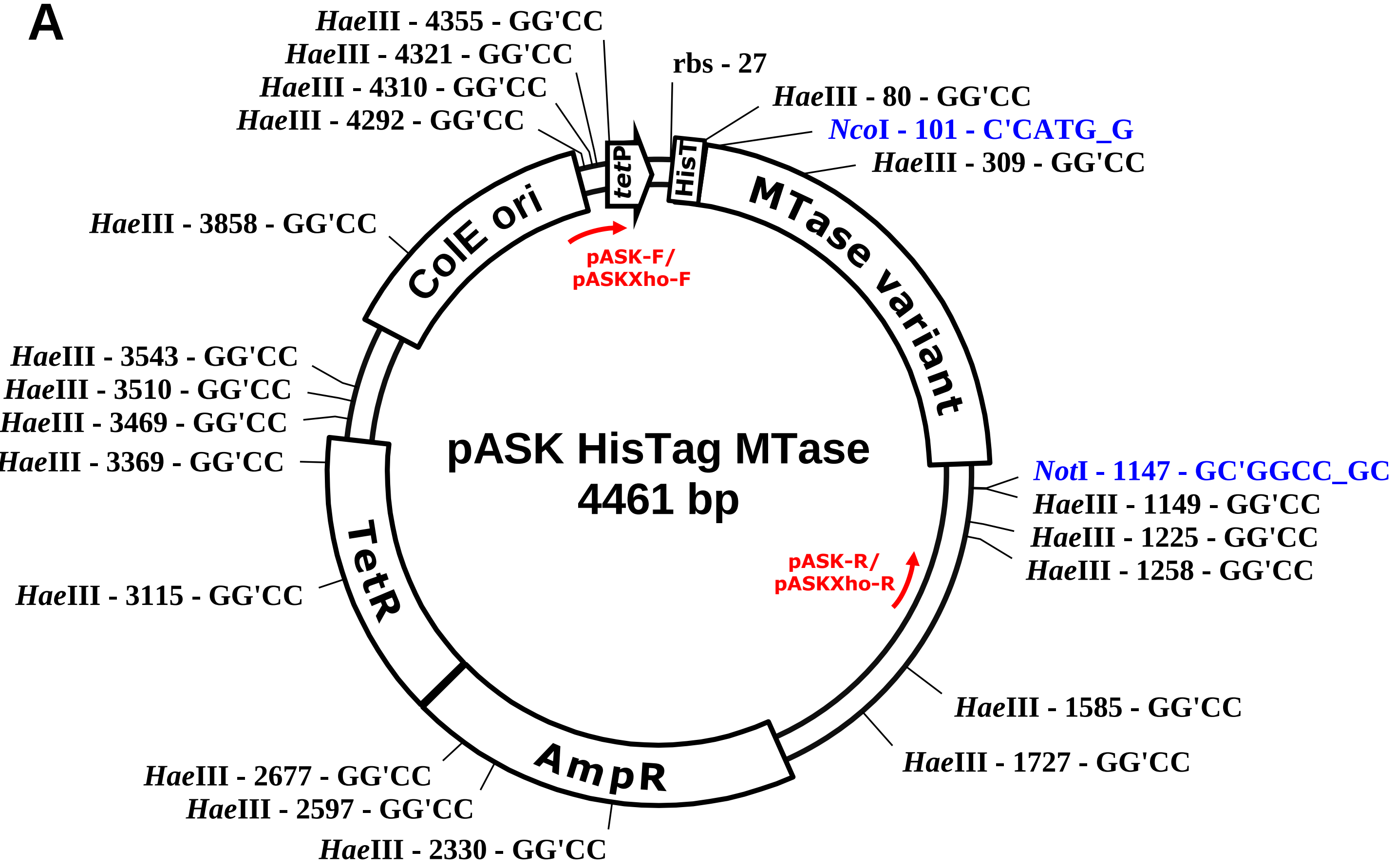

B

Stabilized M.*Hae*III sequence

Nucleotides

ATGGGCAGCAGCCATCATCATCACCATCATAGCAGCGGCCTGGTGCCGCGCGGCAGCTCCATGGCAAATTTAATTA
GTCTTTTTTCAGGTGCAGGGGGATTAGATCTTGGCTTCCAAAAAGCAGGATTTCGTATTATTGCCGCAAATGAATAT
GATAAATCAATATGGAAAACATATGAAAGTAATCATTCAGCTAAATTGATCAAAGGCGATATATCTAAAATTTCTT
CAGATGAATTCCCTAAGTGTGATGGAATTATTGGGGGGCCGCCTTGTCAATCTTGGAGTGAGGGGGGATCTCTTAG
AGGAATTGATGATCCTCGGGGCAAACTTTTTTATGAATATATTCGGATTTTAAAACAAAAAAAACCAAAATTCTTTC
TTGCCGAAAACGTTAAAGGAATGCTGGCTCAGCGTCATAATAAGGCTGTTCAAGAATTTATCCAAGAATTTGATAA
TGCTGGATATGATGTCCATATTATTTTGCTTAATGCGAATGATTATGGTGTAGCTCAAGATAGAAAACGTGTTTTTT
ATATTGGTTTTAGAAAAGAGTTAAATATAAATTATCTTCCACCCATTCCACATTTGATAAAGCCAACATTGAAGGAC
GTCATTTGGGATCTTAAGGATAATCCAATTCCAGCTTTAGATAAAAATAAAACAAATGGTAATAAATGTATTTATCC
TAATCACGAATATTTTATAGGATCATATTCAACAATTTTTATGAGTAGAAATCGTGTTAGACAATGGAATGAACCAG
CATTCACAGTTCAAGCATCTGGACGACAATGTCAATTACACCCCCAAGCTCCTGTAATGTTAAAAGTTAGTAAAAAT
TTAAATAAATTTGTTGAGGGAAAAGAACATTTATATCGCAGACTAACGGTTCGGGAATGTGCACGAGTGCAAGGAT
TTCCAGATGATTTTATTTTTCATTACGAAAGTTTGAATGATGGTTATAAAATGATCGGTAACGCTGTGCCTGTAAATT
TAGCATATGAAATAGCTAAGACAATAAAATCTGCATTGGAAATTCGTAAAGGTAATTAG

Amino acids:

MGSSHHHHHHSSGLVPRGSSMA**NLISLFSGAGGLDLGFQKAGFRIIAANEYDKSIWKTYESNHSAKLIKGDISKISSDE
FPKCDGIIGGPPCQSWSEGGSLRGIDDPRGKLFYEYIRILKQKKPKFFLAENVKGMLAQRHNKAVQEFIQEFDNAG
YDVHIILLNANDYGVAQDRKRVFYIGFRKELNINYLPPIPHLIKPTLKDVIWDLKDNPIPALDKNKTNGNKCIYPNH
EYFIGSYSTIFMSRNRVRQWNEPAFTVQASGRQCQLHPQAPVMLKVSKNLNKFVEGKEHLYRRLTVRECARVQG
FPDDFIFHYESLNDGYKMIGNAVPVNLAYEIAKTIKSALEIRKGN**

C

HA-tag:

CTC	GAG	GGA	GGG	TAC	CCA	TAC	GAT	GTT	CCA	GAT	TAC	GCT	TAG
L	E	G	G	Y	P	Y	D	V	P	D	Y	A	*
