# Supplementary material for: Correlated Occurrence and Bypass of Frame-Shifting Insertion-Deletions (InDels) to Give Functional Proteins
Source: PLoS Genet. 2013 Oct 24;9(10):e1003882. doi: 10.1371/journal.pgen.1003882 (PMC3812077; doi:10.1371/journal.pgen.1003882)
Supplement: Table S1 — InDels within non-repeat sequences. Noted within the sequence column, in bold, is the inserted base, and in bold with strikethrough the deleted one. (PDF) [file pgen.1003882.s011.pdf]

nt	aa	Type	InDel frequency<br>G0 (x10^{-3})	 G17 (x10^{-3})	Sequence
234	78	-A	0.19	0.13	…GGGGGG**~~A~~**TCTCTTA…
		+G			…GGGGGGA**G**TCTCTTA…
237	79	-T	0.00	0.23	…GGGGGGATC**~~T~~**CTTA…
275	92	-A	0.08	0.07	...TTTTTT**~~A~~**TGAA...
298	100	-C	1.80	0.09	…AAA**~~C~~**AAAAAAAACC…
		+G			…AAAC**G**AAAAAAAACC…
		+C			…AAAC**C**AAAAAAAACC…
		+T			…AAAC**T**AAAAAAAACC…
305	102	+T	0.24	0.03	…AAACAAAAAAA**T**ACC…
		+G			…AAACAAAAAAA**G**ACC…
307	103	-C	0.35	0.11	…AAACAAAAAAAA**~~C~~**C…
		+A			…AAACAAAAAAAAC**A**C…
		+T			…AAACAAAAAAAAC**T**C…
418	140	-A	0.09	0.03	…ATATT**~~A~~**TTT...
473	158	-A	0.07	0.16	…TTTTTT**~~A~~**TATT...
		+T			…TTTTTTAT**T**ATT...
871	291	-A	0.11	0.13	…ATTTT**~~A~~**TTTTTC…
