# Supplementary material for: Correlated Occurrence and Bypass of Frame-Shifting Insertion-Deletions (InDels) to Give Functional Proteins
Source: PLoS Genet. 2013 Oct 24;9(10):e1003882. doi: 10.1371/journal.pgen.1003882 (PMC3812077; doi:10.1371/journal.pgen.1003882)
Supplement: Table S2 — Primers list used for library preparation and sequencing. Underlined – the introduced XhoI site. a The priming site is in accordance with the plasmid map (Figure S9). (PDF) [file pgen.1003882.s012.pdf]

**Name**	**Sequence**	**priming site[a]**
pASK-F	GAGTTATTTTACCACTCCCT	4420 forward
pASK-R	CGCAGTAGCGGTAAACG	1318 revers
pASKXho-F	AAA<u>CTCGAG</u>GAGTTATTTTACCACTCCCTATCA	4424 forward
pASKXho-R	AAA<u>CTCGAG</u>CGCAGTAGCGGTAAACG	1318 revers
XhoCtFus-R	AAA<u>CTCGAG</u>ATTACCTTTACGAATTTCCAA	1075 revers
