# Supplementary material for: Correlated Occurrence and Bypass of Frame-Shifting Insertion-Deletions (InDels) to Give Functional Proteins
Source: PLoS Genet. 2013 Oct 24;9(10):e1003882. doi: 10.1371/journal.pgen.1003882 (PMC3812077; doi:10.1371/journal.pgen.1003882)
Supplement: Table S3 — Primers list used for all around PCR generating individually tested InDels (described in Table 3). (PDF) [file pgen.1003882.s013.pdf]

#	Forward primer	Reverse primer	Note
1	AAAAGCAGGATTTCGTATTATTGCC	GGAAGCCAAGATCTAATCCCCC	'A' deletion from 5A repeat at position 57 nt
2	AAAAAAGCAGGATTTCGTATTATTGCC	""	'A' insertion into 5A repeat at position 57 nt
3	GGGGGCCGCCTTGTCAATCTTGG	AATAATTCCATCACACTTAG	'G' deletion from 6G repeat at position 204 nt
4	GGGGGGGCCGCCTTGTCAATCTTGG	""	'G' insertion into 6G repeat at position 204 nt
5	TTTTTATGAATATATTCGGATTT	GTTTGCCCCGAGGATCATCA	'T' deletion from 6T repeat at position 274 nt
6	AAAAAAACCAAAATTCTTTCTTGCCG	GTTTTAAAATCCGAATA	'A' deletion from 8A repeat at position 306 nt
7	AAAAAAAAACCAAAATTCTTTCTTG	""	'A' insertion into 8A repeat at position 306 nt
8	AAAAAAAAAACCAAAATTCTTTCTTG	""	'AA' insertion into 8A repeat at position 306 nt
9	TTTGCTTAATGCGAATGA	TAATATGGACATCATATCCAG	'T' deletion from 4T repeat at position 422 nt
10	TTTTTATATTGGTTTTAGAAAAGAG	CACGTTTTCTATCTTGAG	'T' deletion from 6T repeat at position 472 nt
11	TTTTTTTATATTGGTTTTAGAAAAGAG	""	'T' insertion into 6T repeat at position 472 nt
12	AAAATAAAACAAATGGTAATAAATG	ATCTAAAGCTGGAATTGG	'A' deletion from 5A repeat at position 596 nt
13	AAAAAATAAAACAAATGGTAATAAATG	""	'A' insertion into 5A repeat at position 596 nt
14	TTTTATGAGTAGAAATCGTG	TTGTTGAATATGATCC	'T' deletion from 5T repeat at position 666 nt
15	CCCCAAGCTCCTGTAATGTTAAA	TGTAATTGACATTGTCGT	'C' deletion from 5C repeat at position 758 nt
16	CCCCCCAAGCTCCTGTAATGTTAAA	""	'C' insertion into 5C repeat at position 748 nt
17	AAAGAACATTTATATCGCAG	CCCTCAACAAATTTATTTA	'A' deletion from 4A repeat at position 804 nt
18	TTTTCATTACGAAAGTTTGAATG	TAAAATCATCTGGAAATCCTTGCA	'T' deletion from 5T repeat at position 876 nt
19	TTTTTTCATTACGAAAGTTTGAATG	""	'T' insertion into 5T repeat at position 876 nt
