# Supplementary material for: Correlated Occurrence and Bypass of Frame-Shifting Insertion-Deletions (InDels) to Give Functional Proteins
Source: PLoS Genet. 2013 Oct 24;9(10):e1003882. doi: 10.1371/journal.pgen.1003882 (PMC3812077; doi:10.1371/journal.pgen.1003882)
Supplement: Figure S6 — Frame shifts in M.HaeIII's gene are bypassed to produce full-length enzyme. M.HaeIII variants carrying the frame shift InDels ant C-terminal HA-tag (variants listed in Table 3) were over-expressed and their supernatants were visualized by Western blotting with monoclonal antibody against HA tag. Arrow on the left represents the expected size of a full length (as the wild-type, ‘WT’, of ∼40 KDa). Inserted – a short exposure snapshot of the wild-type M.HaeIII. Experimental protocol: Cell pellets of over-expressed InDel variants were lysed and analyzed by SDS-PAGE, electroblotted onto nitrocellulose membrane, and blocked (0.5% Tween-20 and 5% non-fat milk in 1×PBS). Tag detection was performed using polyclonal anti-HA rabbit antibodies (Abcam, diluted 1∶5000 in PBS, 0.05% Tween-20 and 5% BSA; overnight at 4°C). Peroxidase conjugated anti-rabbit secondary antibody (Jackson, 1∶10000) (0.05% Tween-20 in PBS; incubated 30 mins at 23°C) and EZ-ECL kit (Pierce) were subsequently applied and the signal was detected. (PDF) [file pgen.1003882.s006.pdf]

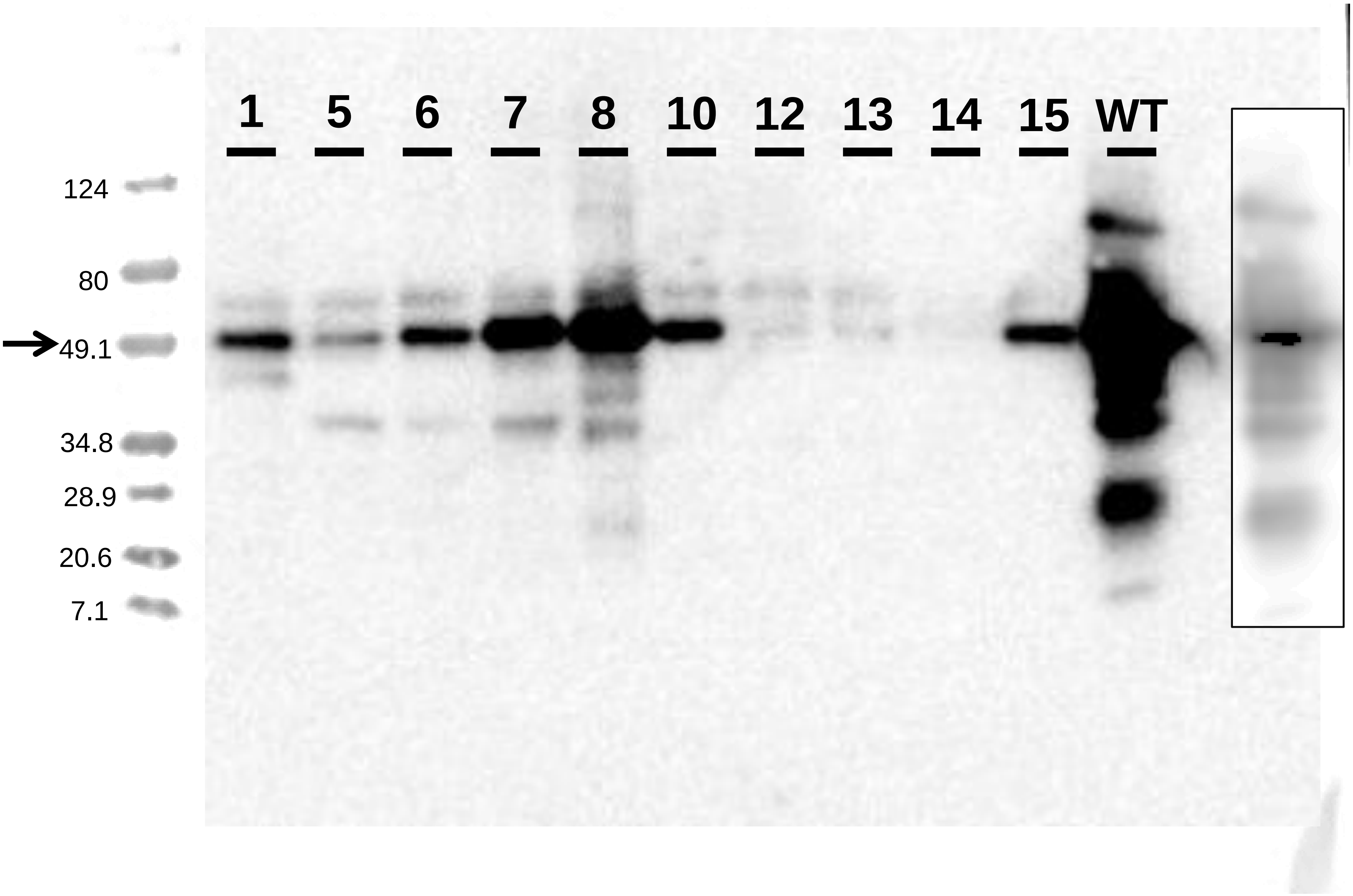
1
5
6
7
8
10
12
13
14
15
WT
124
80
49.1
34.8
28.9
20.6
7.1
